# Application of 2D‐Chromatography System for the Determination of Cosmetic Peptides in Skin Homogenates

**DOI:** 10.1002/jms.70094

**Published:** 2026-07-23

**Authors:** Fosca Errante, Marco Pallecchi, Marta Menicatti, Lisa Giovannelli, Paolo Rovero, Gianluca Bartolucci

**Affiliations:** ^1^ Department of Neurofarba (Department of Neurosciences, Psychology, Drug Research and Child Health) University of Florence Florence Italy; ^2^ Interdepartmental Research Unit of Peptides and Proteins Chemistry and Biology University of Florence Florence Italy

**Keywords:** dermal stability, ion trap mass spectrometry, isomeric peptide analogues, online sample cleanup, scrambled peptide

## Abstract

Peptides are increasingly employed as active ingredients in both therapeutic and cosmeceutical applications due to their high biological specificity, favorable safety profiles, and expanding market relevance. In topical formulations, peptide activity is typically confined to the skin, where widespread dermal proteases may significantly affect their stability and efficacy. Despite the growing use of bioactive peptides in dermatological and cosmeceutical products, robust analytical methodologies for assessing their susceptibility to dermal enzymatic degradation remain limited. In this study, a two‐dimensional HPLC–MS/MS (2D‐HPLC–MS/MS) method based on ion trap detection was developed and validated for the quantitative evaluation of peptide stability in human skin homogenate (HSH). The analytical setup integrates online cleanup with chromatographic separation on a silica‐based pentafluorophenyl (PFP) column, enabling reliable analysis of peptides with different polarity profiles within a single workflow. The system enables direct injection of sample solutions with relatively high organic solvent content, allowing limited sample dilution and preservation of analytical sensitivity. A key feature of the method is the use of isomeric peptide analogues as internal standards, monitored under identical MS/MS conditions as their corresponding analytes. This approach provides effective correction for ionization and fragmentation variability while offering a practical alternative to stable isotope‐labelled standards. The ion trap mass analyzer ensured controlled and reproducible fragmentation behavior, supporting robust quantitative performance. The developed method demonstrated satisfactory linearity, sensitivity, precision, accuracy, and minimal matrix effects. Application to stability studies of three peptides with distinct polarity profiles confirmed its suitability for monitoring degradation kinetics in HSH. Overall, this 2D‐HPLC–MS/MS strategy provides a versatile analytical platform for dermal peptide stability assessment and supports early‐stage screening and preclinical studies of bioactive peptides intended for topical applications.

## 1 Introduction

Peptides are biomolecules composed of amino acids arranged to form sequences of various lengths and compositions. Given their substantial therapeutic potential, promising market prospects, and economic value, peptides are emerging as potent active ingredients with a plethora of application fields. Significant advancements in molecular biology, peptide chemistry, pharmacology, and delivery technologies have catalyzed notable progress in peptide drug discovery, production, and applications [1]. Therapeutics are not the only class of peptides that are having a surge in the last years; in fact, as extensively described in literature, the emerging class of cosmeceutical peptides has increased dynamically [2, 3]. Generally, the route of administration of choice for cosmeceutical peptides is the topical one, and their activity is normally confined to the skin. Despite the great numbers of peptides used to treat skin, there is a lack in terms of analytical methodology to verify their resistance to dermal proteases, which are widely distributed among the human body, including skin [4]. Indeed, this is a crucial point to consider as amino acids linked to each other through amide bonds form peptides that are susceptible to protease activity. Mass spectrometry (MS) is a powerful analytical technique for the identification, characterization, and quantification of molecules and represents one of the most effective analytical tools for peptide studies [5]. MS provides detailed information about the peptide composition of biological samples, enabling insights into cellular processes, supporting their identification as potential biomarkers, and extending the investigation to their interactions with proteins in various biological systems. Previous studies have reported reliable methodologies for quantifying peptide stability against dermal proteases using high‐performance liquid chromatography (HPLC) coupled with tandem mass spectrometry (MS/MS) [6]. However, to broaden the polarity range of peptide candidates and minimize the matrix effects (MEs) of human skin homogenate (HSH) on their detection, a versatile two‐dimensional HPLC (2D‐HPLC) system coupled with an ion trap mass spectrometer was proposed, enabling both enhanced chromatographic separation performance and the exploitation of MS/MS capabilities.

Given the inclusion of polar and hydrophilic peptide candidates within the scope of this study, the nature of secondary interactions between the analytes and the chromatographic system required careful consideration. In this context, chromatographic selectivity could not rely solely on hydrophobic interactions, as typically achieved with conventional C18 stationary phases. For this reason, a silica‐based pentafluorophenyl (PFP) stationary phase was evaluated as an alternative within the proposed 2D‐HPLC system. The PFP column preserves reversed‐phase chromatographic behavior while offering a broader range of interaction mechanisms than conventional C18 phases, including dipole–dipole, hydrogen‐bonding, and π–π interactions. This broader selectivity profile may provide enhanced flexibility for the analysis of peptide candidates exhibiting diverse structural and physicochemical characteristics. Although optimization of the chromatographic system and stationary phase was essential to ensure adequate separation and detection of polar peptide candidates within a complex biological matrix, it also highlighted the need for a robust quantitative strategy capable of accounting for variability introduced throughout the analytical workflow. In this context, the selection of an appropriate internal standard (IS) emerged as a critical aspect of the present study. It is well established that an IS capable of compensating for variability arising from sample preparation, chromatographic separation, ionization efficiency, and MS/MS detection is required to ensure accuracy and reproducibility in quantitative analyses. Ideally, an IS should closely mimic the physicochemical and analytical behavior of the target analyte while remaining clearly distinguishable at the detection stage. In peptide MS/MS studies, this requirement is most commonly fulfilled through the use of stable isotope‐labelled peptides [7, 8, 9]. However, the synthesis of isotope‐labelled peptides is often complex, time‐consuming, and expensive, particularly when large numbers of peptide candidates must be evaluated, making their application impractical in exploratory or screening studies. Considering these limitations, the present study evaluates the use of isomeric peptide analogues as a practical alternative IS strategy for exploratory and screening‐oriented quantitative MS/MS analyses. These analogues may include sequence isomers such as scrambled peptides and reverse peptides, both of which retain the same elemental composition and molecular mass as the parent analyte. Scrambled peptides are commonly synthesized and extensively employed as control compounds to demonstrate that peptide activity arises from a specific amino acid sequence rather than from the mere presence of particular amino acids [10, 11, 12, 13, 14, 15, 16]. Reverse peptides, indeed, maintain the same stereochemistry while presenting the reversion of the sequence [17, 18]. In both cases, the resulting isomeric peptides exhibit physicochemical properties closely related to those of the target analyte, including identical precursor ion masses, while maintaining sufficient structural differences to allow selective MS/MS detection. Their high structural similarity ensures comparable behavior during sample preparation, chromatographic separation, and ionization, whereas their distinct sequence or stereochemical configuration enables unambiguous analytical discrimination. As such, isomeric peptide analogues represent a practical and scalable alternative to stable isotope‐labelled standards for quantitative peptide analysis.

In this study, dermal protease stability was investigated for three peptides with distinct physicochemical properties and analytical roles. AAT11RI and SA1‐III [6, 19, 20, 21, 22] represent new‐generation antiaging peptide candidates differing in size and polarity. In particular, AAT11RI is smaller and more hydrophilic than SA1‐III, allowing the proposed analytical strategy to be evaluated across peptides with distinct chromatographic behaviors. In parallel, palmitoyl‐KTTKS (pal‐KTTKS), a well‐established reference peptide [23, 24, 25] with known susceptibility to dermal proteases [6] and a comparatively higher lipophilicity, was included as a benchmark to assess the proteolytic activity of the skin homogenate.

The combined selection of AAT11RI (highly polar), SA1‐III (intermediate polarity), and palmitoyl‐KTTKS (more lipophilic) enabled a comprehensive assessment of the proposed 2D‐HPLC–MS/MS platform over a broad polarity range. This design was intended to demonstrate the versatility of the 2D‐HPLC configuration employing PFP stationary phases.

Accordingly, distinct 2D‐HPLC–MS/MS methods were developed for the quantitative determination of AAT11RI, SA1‐III, and palmitoyl‐KTTKS following their exposure to HSH containing dermal proteases for up to 24 h.

## 2 Material and Methods

### 2.1 Materials and Chemicals

All the amino acids were from Novabiochem (MerckMillipore; Darmstadt, Germany), palmitic acid ≥ 99% and the Fmoc‐Ser (tBu)‐Wang resin were from Sigma Aldrich (Schnelldorf, Germany), and the Rink‐amide AM resin was from Iris Biotech AG (Marktredwitz, Germany).

Solvents used for peptide synthesis were from Sigma Aldrich (Milan, Italy). HEPES ≥ 99.5% (titration) was from Sigma Aldrich (Schnelldorf, Germany). Reagent A (alkaline copper tartrate solution) and reagent B (Folin reagent for colorimetric assays) of DC‐protein assay were from Bio‐Rad. Acetonitrile (Chromasolv), methanol (Chromasolv), formic acid and ammonium formate (MS grade), and verapamil hydrochloride (analytical standard used as IS for the evaluation of the activity of the skin homogenates) were purchased by Sigma Aldrich (Milan, Italy). Protein concentration was evaluated in a Perkin Elmer Wallac 1420 Victor 2 Multi‐Label Microplate Reader. Ultrapure water 18 MΩ cm (Milli‐Q water) was obtained from Millipore's Simplicity system (Milan, Italy).

The experiments were carried out with a Thermo LTQ ion trap system (Waltham, MA, United States) equipped with Dionex Ultimate 3000 HPLC and an ESI source. Raw data were collected and processed by XCalibur 2.0. The thermostatic oven G‐Therm 015 (F.lli Galli, Milan, Italy) was used to maintain the samples at 37°C during the degradation test, whereas the centrifuge Eppendorf 5415D (Merck, Milan, Italy) was employed to centrifuge the skin homogenate samples.

### 2.2 Peptide Synthesis

pal‐KTTKS (sequence palmitoyl‐Lys‐Thr‐Thr‐Lys‐Ser), SA1‐III (sequence Ac‐Met‐Gly‐Lys‐Val‐Val‐Asn‐Pro‐Thr‐Gln‐Lys‐NH_2_), SA1‐IIIsc (sequence Ac‐Pro‐Val‐Asn‐Thr‐Lys‐Met‐Gln‐Val‐Lys‐Gly‐NH_2_), AAT11RI (sequence Ac‐D‐Asn‐D‐Val‐D‐Val‐D‐Lys‐NH_2_), and AAT11‐allD (sequence Ac‐D‐Lys‐D‐Val‐D‐Val‐D‐Asn‐NH_2_) were prepared by solid‐phase peptide synthesis following previously described protocols [19, 21]. Briefly, synthesis was performed manually on Fmoc‐Ser (tBu)‐Wang resin with a 0.42 mmol/g loading (pal‐KTTKS) and Rink‐amide AM resin with a 0.48 mmol/g loading (SA1‐III, SA1‐IIIsc, AAT11RI, and AAT11‐allD) using the 9‐fluorenylmethoxycarbonyl/tert‐butyl (Fmoc/tBu) strategy. In the case of pal‐KTTKS, the conjugation of palmitic acid to the peptide N‐terminal was achieved on resin using normal acid coupling condition. SA1‐III, SA1‐IIIsc, AAT11RI and AAT11‐allD were acetylated at the N‐terminal. The cleavage was performed in acid condition according to the resin we used. In particular, a mixture of 95% v/v TFA (trifluoroacetic acid), 2.5% v/v Milli‐Q H_2_O, and 2.5% v/v TIS (triisopropylsilane) was employed for peptides pal‐KTTKS, AAT11RI, and AAT11‐allD and a mixture of 92.5% v/v TFA, 2.5% v/v EDT (ethane‐1,2‐dithiol), 2.5 v/v TIS, and 1% v/v Milli‐Q H_2_O was employed for peptides SA1‐III and SA1‐IIIsc due to their methionine residue. After the cleavage, peptides were purified by flash chromatography followed by semi‐preparative chromatography (purification details are reported in Table ST1). The characterization was made by HPLC–MS.

### 2.3 Preparation of Skin Homogenates

Specimens of human skin were taken from a patient (60 years‐old women) subjected to breast surgery at Careggi University Hospital, Florence. Each specimen was deprived of adherent fat and cut into pieces of the same size (0.3 cm^2^). Those pieces were put three by three in 1.5 mL Eppendorf tubes containing 1 mL of ultrapure water, kept on ice, and homogenized with a POLYTRON PT 1200 E handheld homogenizer (Kinematica Inc) for about 15 min and then centrifuged at 10 000 rpm for 30 min at 4°C. All supernatants were transferred in 15 mL Falcon tubes after filtration with syringe filters 0.2 μm and diluted with HEPES buffer solution (pH 7.4; 10 mM), from here on HEPES solution, to obtain a final volume of 7.5 mL. Homogenates were put at first at −80°C to stop the enzymatic activity and after 2 h were moved and stored at −20°C.

### 2.4 Protein Concentration Assay

Total protein concentration was measured in HSHs and was performed according to the manufacturer's instructions. The assay was performed in 96‐mw, adding in sequence: samples, 25 μL reagent A and 200 μL reagent B, which are respectively copper tartrate and Folin reagent from Bio‐Rad (Milan, Italy) kit (DC Protein Assay). After incubation of the plate (37°C, 20 min), absorbance values were determined at 490 nm in a Victor 2 Multi‐Label Microplate Reader.

### 2.5 Standard Solutions

Stock solutions of verapamil (IS1) were prepared at a concentration of 1 mg mL^−1^ in acetonitrile. Stock solutions of pal‐KTTKS, SA1‐III, SA1‐IIIsc (IS2), AAT11RI, and AAT11‐allD (IS3) were prepared at 1 mg mL^−1^ in Milli‐Q water:methanol (50:50, v/v) and stored at 4°C. Working solutions of palmitoyl‐KTTKS, SA1‐III, and AAT11RI (spike solutions 1, 2, and 3 respectively) were freshly prepared by diluting the corresponding stock solutions to a final concentration of 5 μM in Milli‐Q water. IS working solutions were prepared in acetonitrile at concentrations of 0.07 μM for IS1 and 1.67 μM for both IS2 and IS3. Calibration curves for pal‐KTTKS, SA1‐III, and AAT11RI were obtained by preparing three independent series of calibration solutions, each using the corresponding spike solution and IS (IS1, IS2, and IS3, respectively). For each analyte, appropriate aliquots of the respective spike solution were transferred into plastic autosampler vials, followed by the addition of 150 μL of the corresponding IS solution and dilution to a final volume of 500 μL with 10 mM formic acid solution. For all three analytes, the calibration series covered the same concentration range, yielding final analyte concentrations of 0.05, 0.10, 0.20, 0.40, and 0.50 μM. For each calibration series, a blank solution was prepared following the same procedure but without the analyte in order to evaluate potential interference of the IS with the analyte signal. To evaluate the precision and accuracy of analyte quantification, new sets of standard solutions were prepared at three concentration levels (low, medium, and high), corresponding to 0.05, 0.10, and 0.50 μM, respectively, following the same preparation procedure used for the calibration solutions. These solutions were independently prepared in six replicates starting directly from the respective stock solutions, using working solutions distinct from those employed for the calibration curves.

### 2.6 Chromatographic System (2D‐HPLC)

**FIGURE 1:**
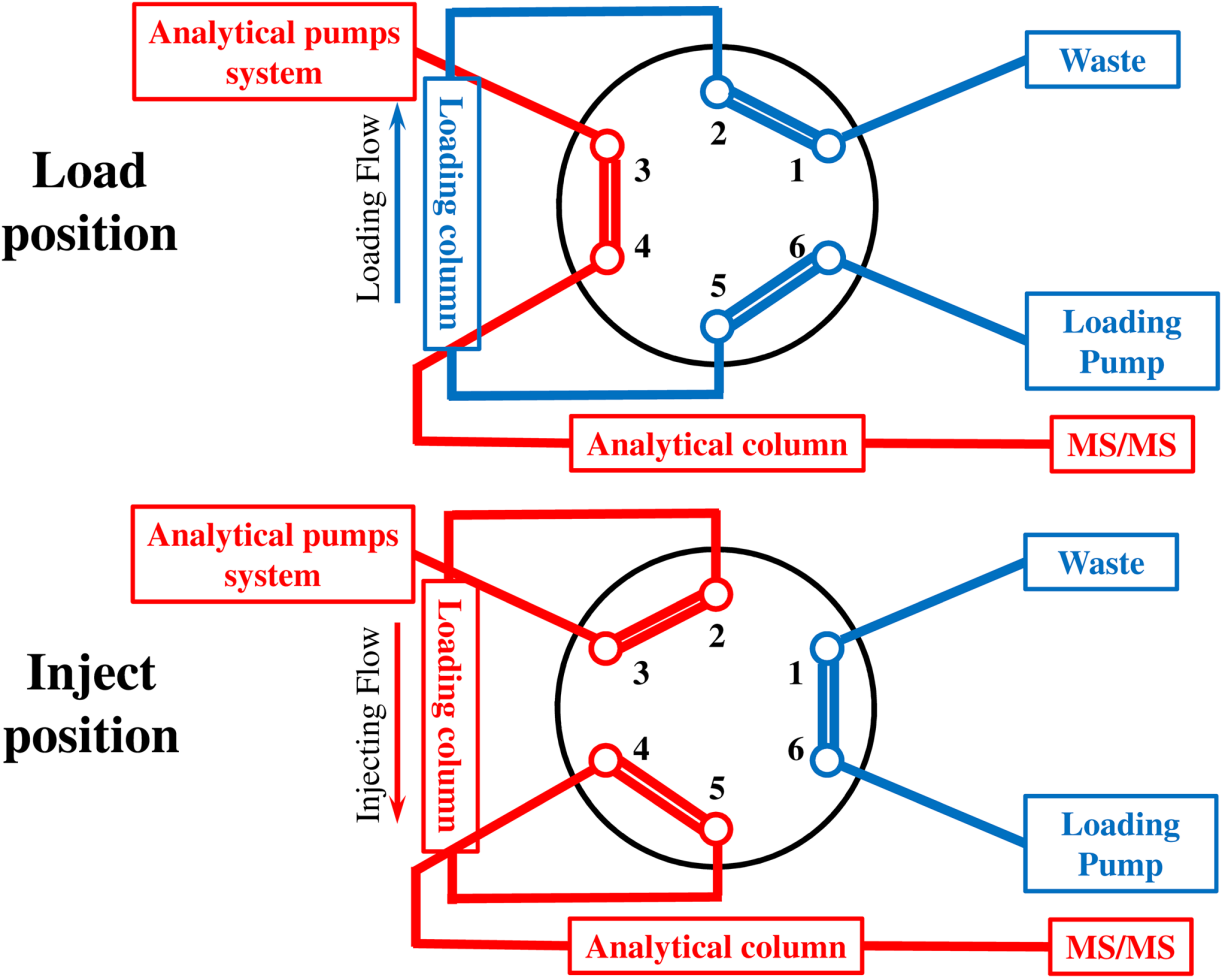
Scheme of 2D‐HPLC system.

The chromatographic system employed for sample analysis was a Dionex Ultimate 3000 (Thermo Fischer Scientific, Waltham, MA, United States) configured as a two‐dimensional HPLC (2D‐HPLC) system and equipped with three pumps and a six‐port switching valve. The system was assembled around the six‐port valve, which enabled transfer between the first and second separation dimensions. In the first dimension (load position), samples were injected under isocratic conditions onto a loading column via the loading pump. During this step, the loading column retained the analytes of interest, whereas non‐retained components, primarily more hydrophilic compounds, were directed to waste. After valve switching to the inject position, the retained components were eluted in a counterflow mode by the analytical pumps and transferred to the chromatographic column representing the second separation dimension. The overall 2D‐HPLC setup and workflow are schematically illustrated in Figure 1.

Sample injection, analyte transfer between the two chromatographic dimensions, and subsequent separation were achieved using three distinct solvent lines. Solvents A and B, managed by the analytical pump system, consisted of formic acid (20 mM) and ammonium formate (5 mM) in Milli‐Q water (solvent A) and in methanol (solvent B), respectively. Solvent C, delivered by the loading pump, consisted of Milli‐Q water:methanol (90:10, v/v) supplemented with 20 mM formic acid and 5 mM ammonium formate. The chromatographic program began with the injection of 20 μL of sample solution, which was transferred by the loading pump onto a Discovery HS F5 Supelguard cartridge (20 × 2.1 mm, 3 μm; Merck Life Science S.r.l., Milan, Italy) serving as the loading column. The sample was retained for 3 min under isocratic conditions using solvent C at a flow rate of 0.50 mL min^−1^. The loading time was optimized using AAT11RI as a probe peptide, selected as the most polar and therefore the most critical analyte in terms of retention on the loading column in the first chromatographic dimension. A 5‐μM standard solution of AAT11RI (spike solution 3) was analyzed under 2D‐HPLC–MS conditions at increasing loading times (0.5, 1.0, 2.0, 2.5, 3.0, 4.0, and 5.0 min). For each loading time, samples were analyzed in triplicate, and the analyte signal was monitored by full‐scan MS detection in the m/z range 250–1200 to evaluate chromatographic peak area as an indicator of analyte recovery. Based on these experiments, a loading time of 3.0 min was selected as the optimal compromise between effective retention of the highly polar peptide and removal of non‐retained matrix components. Following the loading step, the six‐port valve was switched (inject position) to enable counterflow elution of the retained compounds, which were transferred to a Phenomenex Luna PFP(2) analytical column (50 × 2.1 mm, 3 μm; Phenomenex, Bologna, Italy) for second‐dimension separation. The elution gradient of the second chromatographic dimension, operated at a flow rate of 0.25 mL min^−1^, was programmed as follows: Solvent B was maintained at 10% for the first 3 min, then increased to 90% over 4 min and held at this composition for 5 min, after which the initial conditions were restored. The total run time was 15 min. The temperature of loading and analytical columns was maintained at 25°C.

### 2.7 MS and MS/MS Methods

Mass spectrometric analysis was performed in full‐scan mode over the m/z range 250–1200 in positive‐ion mode using an LTQ‐Orbitrap XL (Thermo Fischer Scientific, Waltham, MA, United States) mass spectrometer operated with ion trap parameters obtained from routine instrument tuning. Electrospray ionization (ESI) source parameters were optimized by direct infusion of a 5‐μM SA1‐III solution prepared in Milli‐Q water using a syringe pump at a flow rate of 5 μL min^−1^. The resulting ESI conditions were achieved using the following settings: sheath gas at 35 arbitrary units (a.u.), auxiliary 10 a.u. and sweep gases at 5 a.u., source voltage of 5 kV, capillary voltage of 45 V, capillary temperature of 275°C, and tube lens voltage of 60 V. For MS/MS method development, energy‐resolved tandem mass spectrometry (ERMS) experiments were performed to investigate the fragmentation behavior of the precursor ion of each studied compound and to determine the optimal excitation energy for the final method operated in product ion scan (PIS) mode over the m/z range 250–1200. ERMS experiments were carried out by direct infusion of individual analyte solutions (5 μM) using a syringe pump at a flow rate of 5 μL min^−1^. ERMS experiments consisted of a series of PIS analyses performed on the same compound, with the selected precursor ion subjected to progressively increasing normalized excitation energies, expressed in arbitrary units (a.u.) and ranging from 5 to 30, while maintaining constant isolation width (3.0 m/z), excitation time (50 ms), and *q* value (0.25). Following analysis of ERMS data, MS/MS acquisition for quantitative analysis was performed using three compound‐specific methods, as reported in Table 1.

**TABLE 1:** Sum up of the parameters set up for the experiments.

MS/MS method	Compounds monitored	Time segment (min.)	Precursor ion (m/z)	Excit. energy (a.u.)	Product ion range (m/z)
A	Verapamil (IS1) pal‐KTTKS	0.00:8.30 8:30–12.00	455.1 802.0	25 20	125–470 220–810
B	SA1‐IIIsc. (IS2) SA1‐III	No time segment	571.5	20	250–1050
C	AAT11‐allD (IS3) AAT11RI	No time segment	500.1	20	140–520

### 2.8 Sample Preparation

Three experimental protocols (Experiments A–C) were carried out to evaluate peptide stability in HSH in the presence of dermal proteases. For each experiment, 50 μL of the corresponding spike solution were mixed with 50 μL of HSH, yielding an initial peptide concentration of 2.5 μM in the incubation mixture. Samples were incubated at 37°C for 0, 8, and 24 h. After incubation, 150 μL of the appropriate IS solution were added, inducing protein denaturation and precipitation; then, the sample was centrifuged for 3 min at 10000 rpm and the supernatants were transferred to plastic autosampler vials and further diluted with 250 μL of 10 mM formic acid solution. This procedure resulted in an overall 1:10 dilution of the original HSH matrix prior to injection into the 2D‐HPLC–MS/MS system. Consequently, a peptide concentration of 2.5 μM in the incubation mixture corresponded to a final analytical concentration of 0.5 μM. The resulting sample solution consisted of an aqueous mixture containing approximately 30% (v/v) acetonitrile, a composition compatible with the selected chromatographic loading conditions. Experiment A was performed using pal‐KTTKS and IS1 to assess the proteolytic activity of the homogenate. Experiments B and C were conducted using SA1‐III/IS2 and AAT11RI/IS3, respectively. Each experiment was analyzed using the corresponding 2D‐HPLC–MS/MS method.

In addition, a fourth experiment was performed to evaluate the ME associated with the proposed 2D‐HPLC–MS/MS method and to assess the effectiveness of the online cleanup provided by the first chromatographic dimension. The ME was evaluated for each analyte using a post‐extraction addition approach. For each peptide/IS pair, two sets of samples (Set A and Set B) were prepared in triplicate. Set A (neat solution) was obtained by mixing 50 μL of the corresponding spike solution with 150 μL of the appropriate IS solution. Set B (matrix‐matched solution) was prepared by mixing 50 μL of HSH with 150 μL of the corresponding IS solution; after centrifugation and collection of the supernatant, 50 μL of the respective spike solution was added. The resulting solutions were transferred to autosampler vials and diluted by the addition of 0.25 mL of 10 mM formic acid solution prior to analysis.

### 2.9 Validation of HPLC–MS/MS Methods

Calibration curves for each peptide were constructed by plotting the peak area ratios (PAR) between the analyte and the corresponding IS quantifier ions versus the nominal concentrations of the calibration solutions. Linear regression analysis was applied to determine the best‐fitting calibration function. Limit of detection (LOD) for each analyte was estimated using the standard deviation (SD) of the response and slope approach, with response variability derived from the SD of the y‐intercepts of the calibration curves [26]. ME was evaluated by comparing the absolute peak areas of each analyte obtained from the sets of Samples A and B, prepared as described in Section 2.8, according to the equations reported below:

ME%=1−AreaanalyteSetBAreaanalyteSetA*100

Precision and accuracy of the 2D‐HPLC–MS/MS method were assessed by analyzing the standard solutions at three concentration levels (low, medium, and high) prepared as described in Section 2.5. Accuracy was expressed as recovery (%) by comparing the measured concentrations with the nominal values, whereas precision was evaluated as the SD of replicate measurements at each concentration level.

## 3 Results and Discussion

### 3.1 Peptide Characterization

**FIGURE 2:**
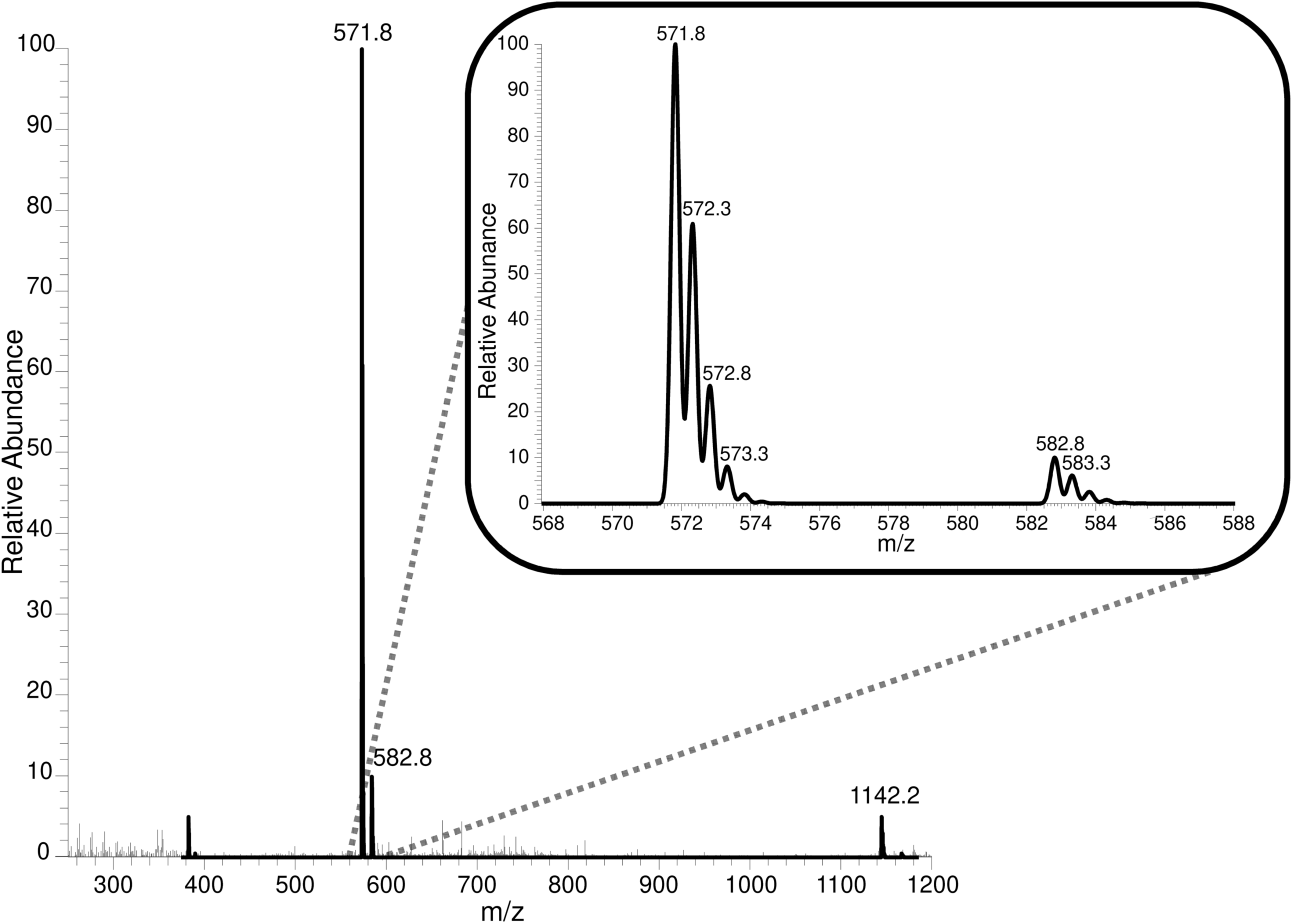
MS spectrum of SA1‐III.

The full‐scan mass spectrum of SA1‐III is shown in Figure 2, whereas the spectra of the other studied peptides and ISs are provided in Figures SF1–SF5.

The mass spectra of SA1‐III and its scrambled analogue exhibited a similar ionization pattern, characterized by an abundant signal at m/z 571.8 corresponding to the doubly charged [M + 2H]^2+^ ion. Additional minor peaks were observed and assigned to the singly charged [M + H]^+^ ion at m/z 1142 and to the sodiated doubly charged [M + H + Na]^2+^ species at m/z 582.8. In contrast, the mass spectrum of pal‐KTTKS showed two predominant ionic species: the doubly charged ion at m/z 401.8 ([M + 2H]^2+^) and the singly charged ion at m/z 802.4 ([M + H]^+^). Notably, the singly charged species was the most abundant signal, exhibiting an intensity approximately twice that of the corresponding doubly charged ion. As observed for SA1‐III, AAT11RI and its reverse analogue exhibited a comparable ionization profile; however, in this case, only singly charged ions were detected. The corresponding spectra (Figures SF4–SF5) showed two main signals: the protonated molecular ion [M + H]^+^ at m/z 500.1 and a less abundant peak at m/z 522.1, assigned to the sodiated adduct [M + Na]^+^.

Accordingly, ERMS experiments were carried out on the studied compounds to investigate their collision‐induced dissociation (CID) behavior for MS/MS method development. Doubly charged precursor ions ([M + 2H]^2+^) were selected for SA1‐III and its scrambled analogue, whereas singly charged precursor ions ([M + H]^+^) were employed for pal‐KTTKS, verapamil, AAT11RI, and its reverse analogue.

### 3.2 ERMS Experiments

**FIGURE 3:**
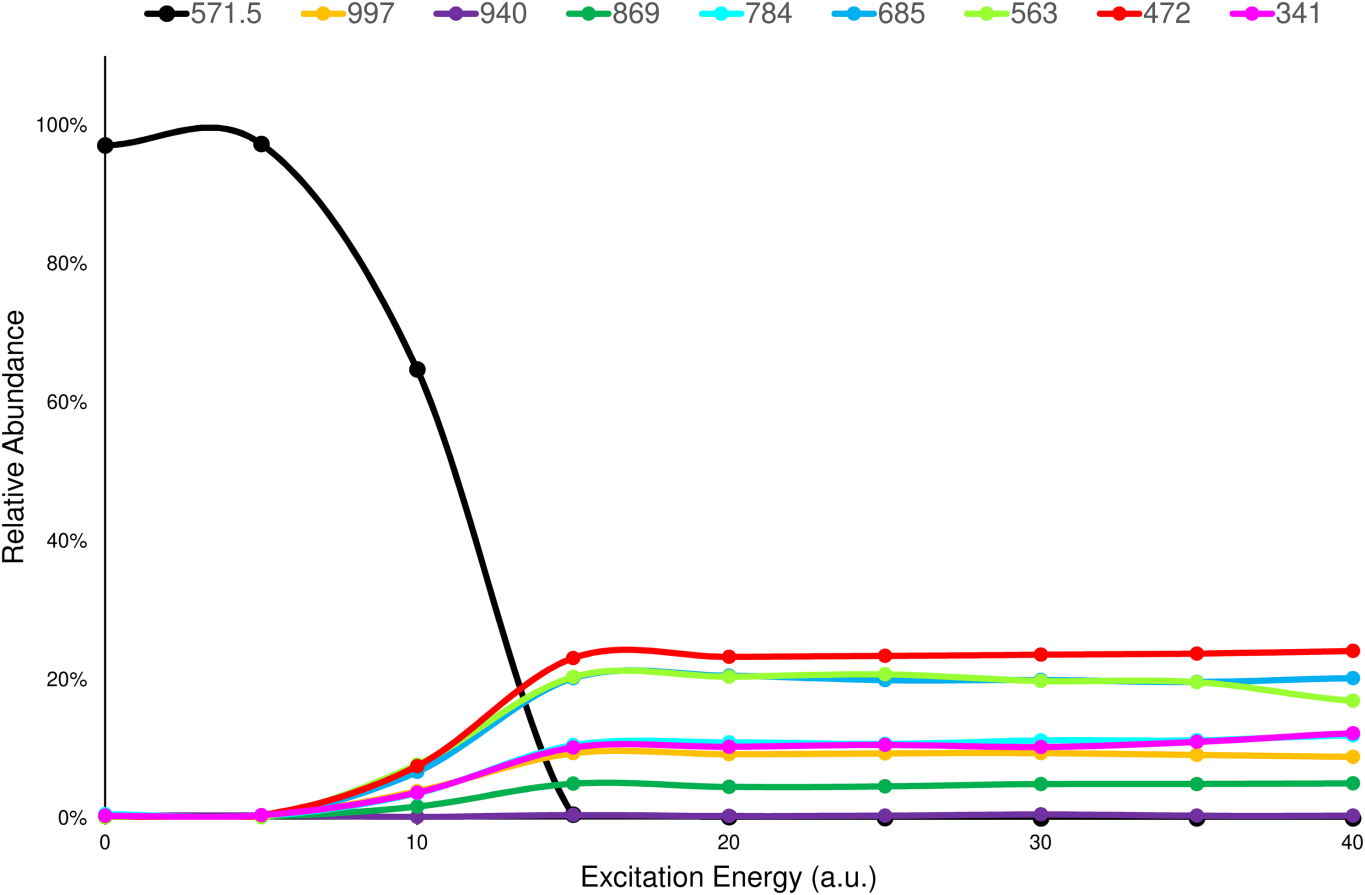
Collision breakdown plot of SA1‐III.

The collected ERMS data were used to generate collision breakdown plots for the studied compounds by plotting the relative intensities of the precursor and corresponding fragment ions as a function of the applied excitation amplitude. The collision breakdown plot of SA1‐III is shown in Figure 3 as an example, whereas the corresponding plots for the other compounds are reported in Figures SF6–SF10.

The collision breakdown plots obtained for the studied compounds showed consistent behavior, characterized by a progressive decrease in precursor ion intensity with increasing excitation amplitude and the concomitant formation of product ions, as represented in the collision breakdown plot of SA1‐III. Interestingly, although the intensity of the precursor ions decreases, the relative composition and abundance of the product ions remain essentially unchanged over the excitation range studied. This behavior is characteristic of ion trap MS/MS experiments and reflects the effective control over the energy transferred during the CID process. Under these conditions, fragmentation pathways are well defined and extensive secondary fragmentation is minimized, resulting in stable and reproducible product ion distributions. The absence of significant secondary dissociation processes leads to an overall higher and more consistent yield of product ions compared with other tandem mass analyzers, such as triple quadrupole instruments, where product ion intensities may vary more markedly as a function of collision energy. This feature of the ion trap platform is particularly advantageous for quantitative peptide analysis, as it ensures robust signal generation and consistent response across a wide range of excitation conditions.

**FIGURE 4:**
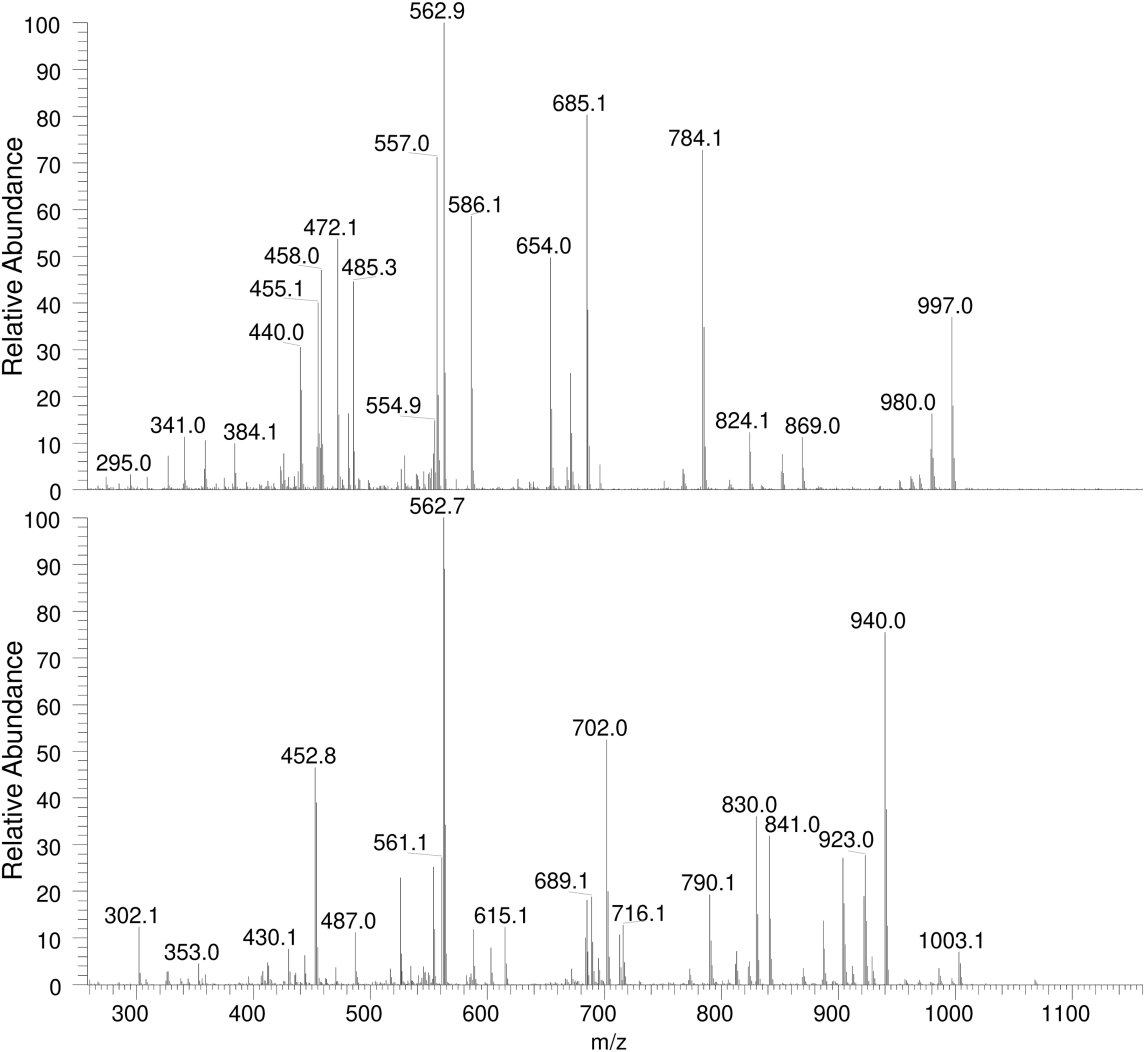
Comparison between MS/MS spectra of SA1‐III (top) and SA1‐IIIsc (bottom).

**FIGURE 5:**
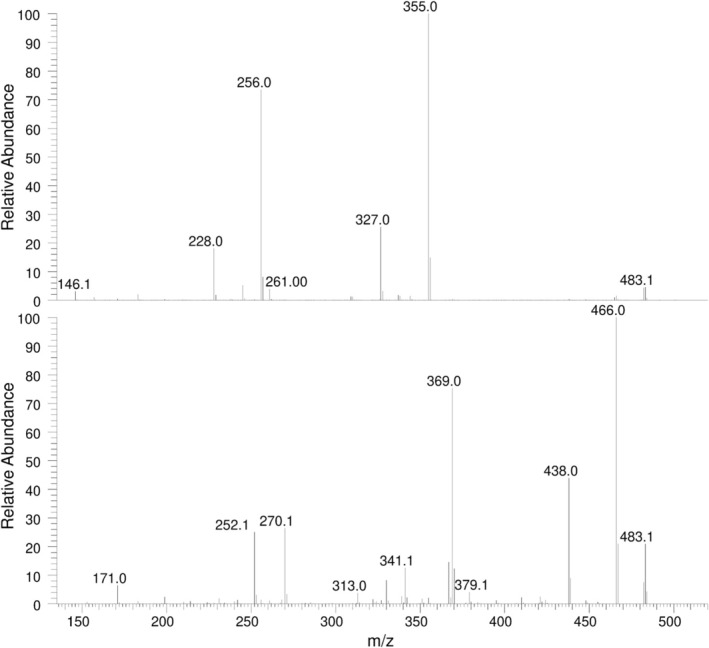
Comparison between the MS/MS spectra of AAT11RI (top) and AAT11‐allD (bottom).

On the basis of the ERMS experiments, optimal excitation amplitudes (ExA) were selected for each compound to ensure efficient and reproducible CID. An excitation amplitude of 20 a.u. was adopted for pal‐KTTKS, SA1‐III and its scrambled analogue, AAT11RI, and its reverse analogue, whereas a higher excitation amplitude (ExA = 25 a.u.) was selected for verapamil. Comparative MS/MS spectra acquired at the selected excitation amplitudes for SA1‐III and its scrambled analogue, and for AAT11RI and its reverse analogue, are presented in Figures 4 and 5, respectively. The corresponding spectra for pal‐KTTKS and verapamil are provided in Figures SF11–SF12.

The MS/MS spectra of SA1‐III and its scrambled analogue, both generated from the common doubly charged precursor ion at m/z 571.8, display clearly distinct fragmentation patterns. This difference enabled the identification of compound‐specific product ions suitable for quantitative analysis. In particular, the product ion at m/z 997 was selected for SA1‐III, whereas the fragment at m/z 940 was selected for the scrambled peptide, as both ions were among the most intense signals in their respective spectra and were not detected in the MS/MS spectrum of the other compound. It is worth noting that both product ions originate from the loss of the C‐terminal amino acid residue, representing a chemically plausible and highly reproducible fragmentation pathway under ion trap CID conditions.

Although additional intense fragment ions were observed, they were not considered suitable for quantification because they did not provide sufficient specificity to reliably discriminate between the analyte and its IS. In contrast, the selected fragments arise from analogous terminal cleavages yet exhibit distinct m/z values, ensuring the absence of mutual interference and supporting their use as quantifier ions in the proposed MS/MS method. As shown in Figure 5, the MS/MS spectra of AAT11RI and its reverse analogue, generated from the selected singly charged precursor ion, exhibited clearly distinguishable fragmentation patterns.

The product ions at m/z 355 for AAT11RI and at m/z 369 for its reverse analogue were selected for quantitative analysis, as they represented intense and compound‐specific fragments that were not detected in the corresponding spectrum of the paired compound. Despite the presence of additional intense fragment ions, particularly in the spectrum of the reverse analogue, they were not considered suitable for quantification because they did not provide sufficient specificity to reliably discriminate between the analyte and its IS. In contrast, the selected fragments arise from a well‐defined C‐terminal cleavage yet exhibit distinct m/z values, ensuring the absence of mutual interference and supporting their use as quantifier ions in the proposed MS/MS method.

Unlike the peptide/isomeric analogue pairs discussed above, the MS/MS behavior of pal‐KTTKS and its structurally unrelated IS (verapamil or IS1) was also investigated. As shown in Figures SF11 and SF12, both compounds exhibited relatively simple fragmentation patterns dominated by two major product ions. This facilitated the straightforward selection of compound‐specific quantifier and qualifier ions. For pal‐KTTKS, the fragment at m/z 697.1 was selected as the quantifier ion, whereas the ion at m/z 784.1 was used as the qualifier. Likewise, for verapamil, the product ions at m/z 165.0 and 303.0 were selected as quantifier and qualifier ions, respectively.

Overall, for all peptides investigated, the selection of quantifier ions was based on the identification of intense, sequence‐relevant, and compound‐specific fragments, ensuring selective MS/MS detection and reliable discrimination between each analyte and its corresponding IS. The precursor, quantifier, and qualifier ions selected for quantitative analysis are summarized in Table ST2.

### 3.3 Chromatographic Performances

**FIGURE 6:**
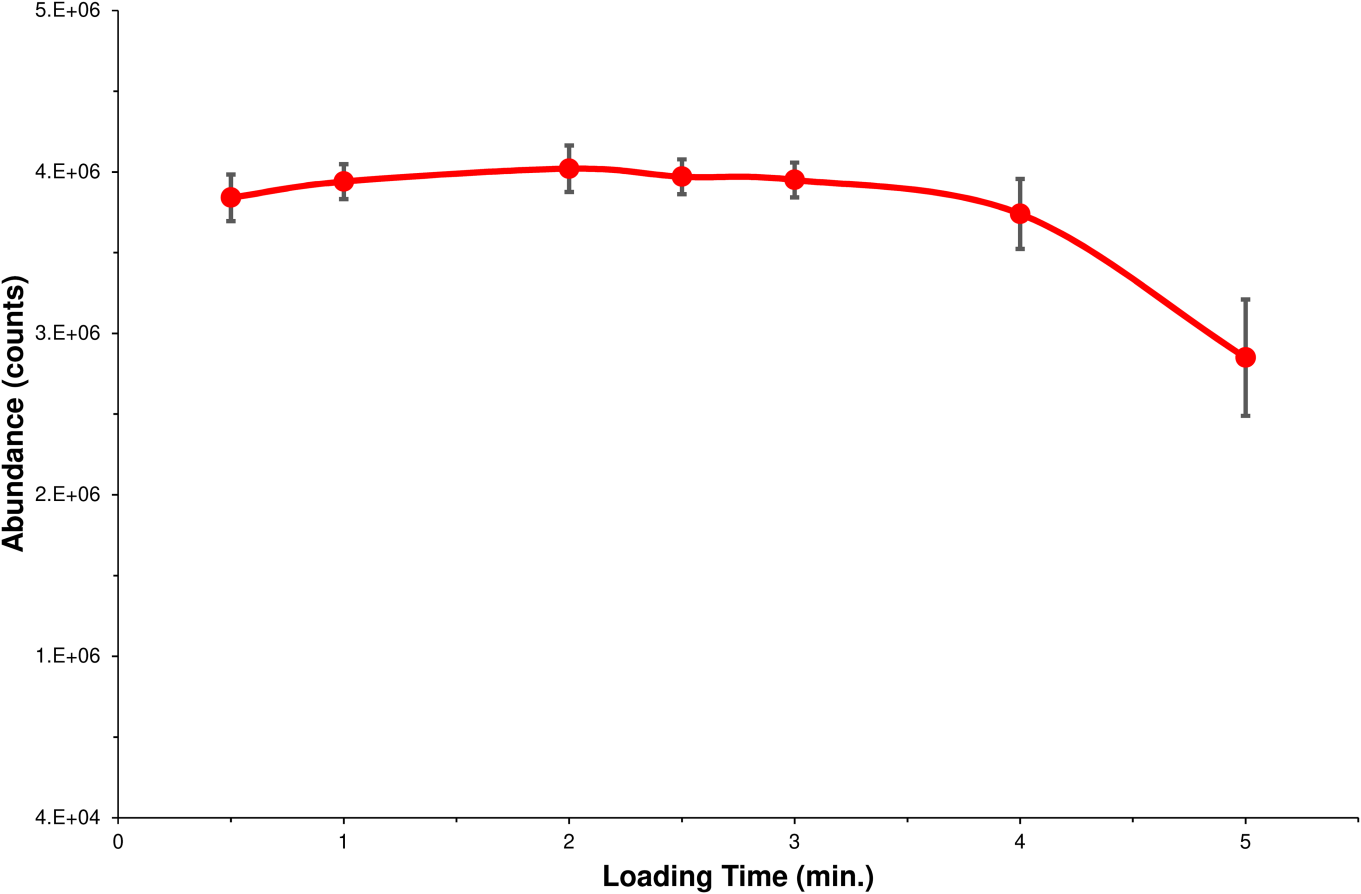
The relationship between chromatographic peak area and loading time for peptide AAT11RI.

The use of a 2D‐HPLC setup in this study was primarily intended to improve sample handling and chromatographic performance for highly polar peptide analytes. Although the first chromatographic dimension mainly served as an online trapping and cleanup step, its role extended beyond simple sample preconcentration, providing significant advantages for the analysis of complex matrices such as HSHs, which are characterized by high concentrations of inorganic salts and other hydrophilic constituents. Under ESI conditions, these species may coelute with the target analytes and negatively affect their ionization efficiency, leading to so‐called “MEs.” In the proposed 2D‐HPLC configuration, sample injection onto the loading column (first chromatographic dimension) enabled the effective removal of non‐retained matrix components, such as inorganic salts, whereas the retained analytes were subsequently eluted in counterflow after the selected loading time and transferred to the analytical column (second chromatographic dimension) for separation and MS/MS detection. Additionally, the involvement of a six‐port valve to enable the samples transfer between the first and the second column resembles a solid‐phase extraction (SPE) procedure, with the advantage of being performed entirely online between the autosampler and the analytical column. The separation of retained components in the second chromatographic dimension was achieved by gradient elution delivered by the analytical pump system, with detection performed by an ion trap MS/MS system. As a result, this configuration provided additional practical advantages. In particular, the online cleanup performed in the first chromatographic dimension enabled the use of a fast sample preparation based on simple protein precipitation, without any requirement to substantially reduce the organic solvent content of the final sample solution. This configuration allowed samples containing relatively high proportions of organic solvent (up to 50%) to be injected without adversely affecting chromatographic performance. Moreover, the system enabled the injection of relatively large sample volumes (in the tens of microliters range), thereby improving overall method sensitivity. Given the critical role of the loading step in balancing matrix cleanup and analyte recovery, the loading time was optimized using AAT11RI as a probe peptide. As shown in Figure 6, the chromatographic peak area of AAT11RI remained essentially constant for loading times between 0.5 and 3.0 min, with comparable SD values across replicates.

**FIGURE 7:**
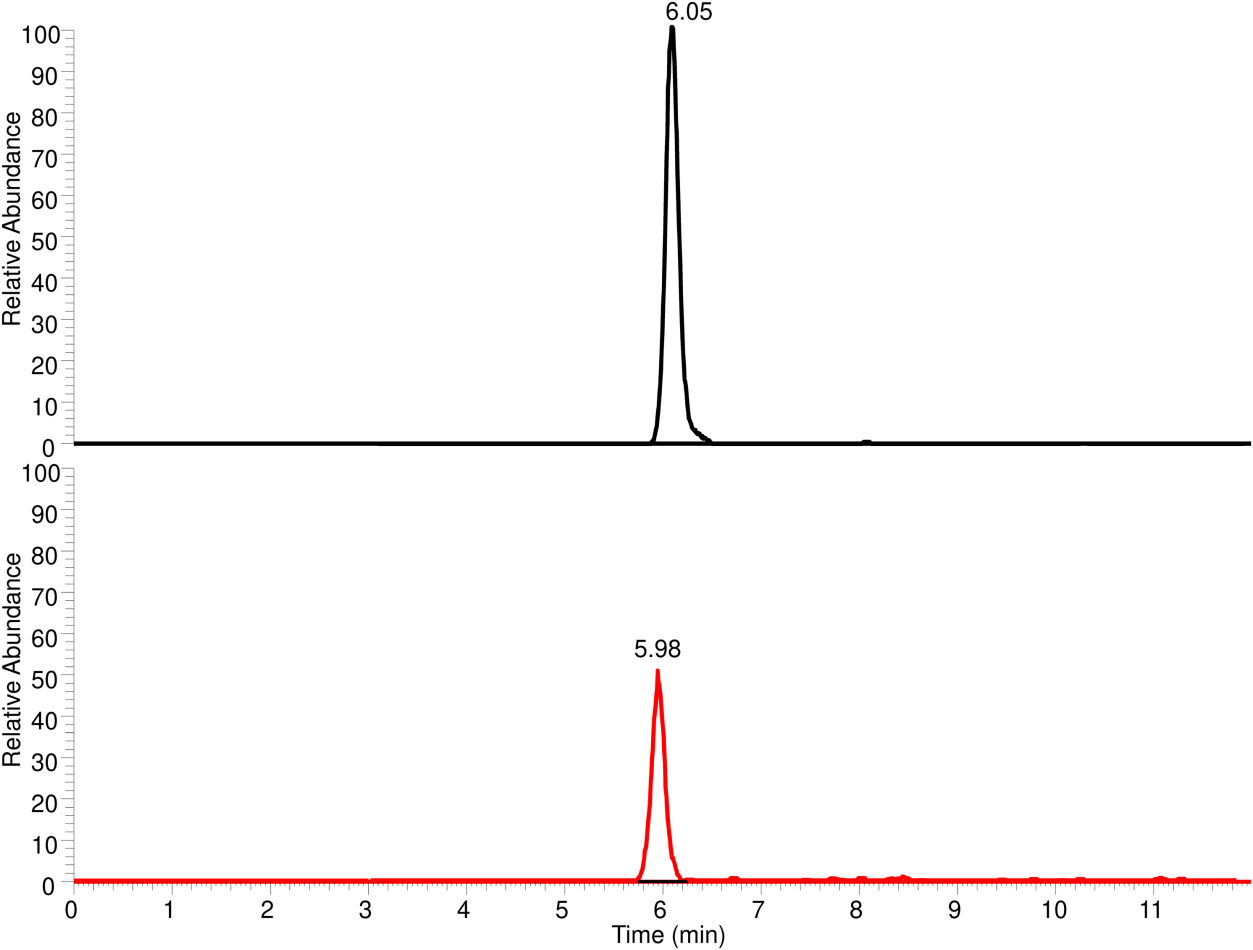
Chromatographic profile of a calibration solution containing AAT11RI and its internal standard (IS3).

Beyond 3.0 min, a progressive decrease in signal intensity was observed, accompanied by an increase in variability, particularly at 5.0 min. These findings indicate that extending the loading time beyond 3.0 min does not improve analyte retention and may instead promote partial loss of the highly polar peptide during the trapping step. Therefore, a loading time of 3.0 min was selected as the optimal compromise between effective matrix cleanup and preservation of analyte recovery in the first chromatographic dimension. Following optimization of the loading step, the elution program of the second chromatographic dimension was optimized to achieve adequate separation between target peptides and respective IS, maintaining acceptable analysis times. The final gradient conditions adopted are described in Section 2.6. Despite the relatively high organic content of both standard and processed sample solutions (approximately 30% acetonitrile, v/v), the proposed 2D‐HPLC configuration ensured effective analyte trapping and satisfactory chromatographic retention. In particular, adequate retention and peak shape were achieved even for the highly hydrophilic peptide AAT11RI, demonstrating the efficiency of the loading column in focusing analytes prior to transfer to the analytical PFP column. These results confirm that the chromatographic setup is compatible with moderately strong injection solvents without compromising separation performance. The resulting chromatographic profiles indicate that complete peak separation between the peptide analytes and their corresponding peptide‐based IS s was not achieved under the optimized conditions. This behavior was observed for both AAT11RI/IS3 and SA1‐III/IS2 pairs. A representative example of the chromatographic overlap between AAT11RI and its reverse analogue is shown in Figure 7, whereas the corresponding chromatographic profile for SA1‐III and its scrambled counterpart is reported in Figure SF14.

Nevertheless, the use of distinct, compound‐specific product ions enabled selective and unambiguous MS/MS detection, ensuring reliable discrimination between analyte and IS signals. The specificity of this approach was further confirmed by the analysis of blank samples prepared in the absence of analyte and containing only the corresponding IS. As shown in Figures SF15–SF16, no detectable signal was observed in the analyte trace under these conditions, confirming the absence of cross‐interference despite partial chromatographic co‐elution. In contrast, the separation between pal‐KTTKS and verapamil (IS1) under the same chromatographic conditions resulted in complete peak separation between the compounds (Figure SF13), confirming the applicability of the optimized elution program to all analyte/IS pairs. However, the use of structurally related peptide analogues (scrambled and reverse forms) as ISs provides an additional methodological advantage. Owing to their isomeric nature and identical precursor ion properties, the analyte and its IS can be monitored under identical MS/MS conditions (precursor ion, excitation amplitude, and acquisition event), without the need to implement separate MS/MS acquisition settings. By contrast, the pal‐KTTKS/verapamil pair requires distinct precursor ions and different MS/MS parameters, which must be applied in separate acquisition events. Consequently, complete chromatographic separation between the two compounds becomes necessary to ensure reliable quantification. This feature simplifies method implementation and enhances acquisition efficiency while maintaining analytical selectivity.

### 3.4 2D‐HPLC–MS/MS Method Performances

Prior to application of the proposed 2D‐HPLC–MS/MS method to dermal stability studies, its analytical performance was evaluated in terms of linearity, sensitivity, precision, accuracy, and ME. Method validation was performed using standard solutions and matrix‐matched samples prepared as described in the Materials and Methods section. The calibration results obtained for both analytes, including linear regression parameters (slope and *y*‐intercept), coefficients of determination (*R* ^2^), and the estimated LOD and limit of quantification (LOQ), are reported in Table ST3. The calibration curves obtained over the concentration range 0.05–0.50 μM exhibited good linearity for all analytes, with coefficients of determination (*R* ^2^) consistently higher than 0.992 (SF17–SF19). The regression parameters demonstrated stable and reproducible detector response across the investigated range, confirming the suitability of the selected IS normalization strategy. The estimated LOD values ranged between 0.02 and 0.03 μM, that is, below the lowest calibration level, whereas the achieved LOQ allowed reliable quantification of the analytes at concentrations as low as 20% of their initial levels in the stability experiments. These results confirm that the proposed 2D‐HPLC–MS/MS method is suitable for monitoring peptide degradation over time in HSH without requiring additional pre‐concentration steps. The results obtained for accuracy, precision, and ME for all analytes are summarized in Table 2.

**TABLE 2:** Results of accuracy at three concentration levels, precision, and matrix effects (ME) of studied peptides.

	Low level (recovery% ± SD)	Mid level (recovery% ± SD)	High level (recovery% ± SD)	ME (value% ± SD)
pal‐KTTKS	84% ± 13%	81% ± 13%	93% ± 11%	2% ± 7%
SA1‐III	107% ± 11%	89% ± 10%	105% ± 10%	2% ± 10%
AAT11RI	112% ± 1%	97% ± 5%	103% ± 9%	4% ± 9%

Accuracy, expressed as percentage recovery, ranged from 81% to 112% across all analytes and concentration levels, indicating satisfactory trueness of the method. Precision, assessed as SD of replicate measurements, ranged from 1% to 13%, demonstrating good repeatability over the tested concentration interval. The observed variability remained well within commonly accepted bioanalytical criteria for quantitative methods. ME, determined using a post‐extraction addition approach, were limited, ranging from 2% to 4%, with SD values comparable with those obtained for precision experiments. These results indicate minimal ion suppression or enhancement from HSH components. Overall, the combined accuracy, precision, and ME data confirm that the proposed 2D‐HPLC–MS/MS method provides reliable and reproducible quantification within the investigated concentration range. The online cleanup enabled by the two‐dimensional chromatographic configuration effectively mitigates matrix‐related interference, ensuring quantitative determination of the peptides without significant influence from the biological matrix.

### 3.5 Peptide Stability Study

**FIGURE 8:**
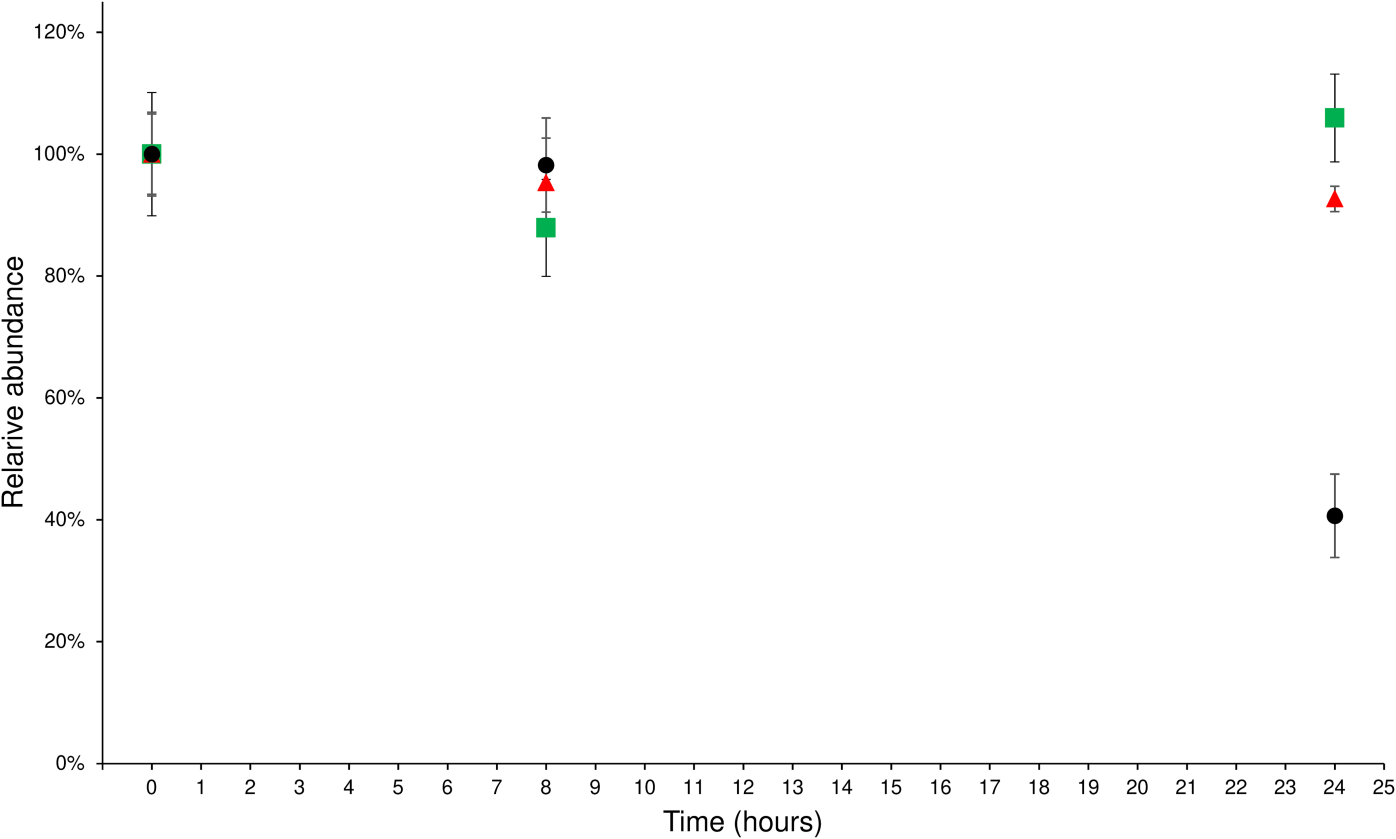
Comparison of the enzymatic degradation profiles of pal‐KTTKS (black circles), SA1‐III (red triangles), and AAT11RI (green squares) in human skin homogenate (HSH). Data are expressed as percentage of the initial concentration (t₀). Error bars represent ± 2 × SD.

The validated 2D‐HPLC–MS/MS method was applied to investigate the stability of AAT11RI, SA1‐III, and pal‐KTTKS in HSH under the experimental conditions described in the Materials and Methods section. Peptide concentrations were monitored over time and expressed as a percentage of the initial concentration (*t*_0_), allowing direct comparison of degradation profiles. Distinct stability behaviors were observed among the three peptides, reflecting their different susceptibility to enzymatic hydrolysis in the HSH matrix (SF20–SF22). Pal‐KTTKS was included in the study as a reference peptide with known susceptibility to proteolytic degradation. Its degradation profile therefore also served to verify the enzymatic competence of the HSH preparation employed in this work. After an initial phase (up to approximately 8 h) characterized by comparable trends for all peptides, pal‐KTTKS exhibited a pronounced decline in concentration, reaching approximately 40% of its initial value at 24 h (Figure 8).

This behavior confirms both its susceptibility to hydrolytic processes and the preserved enzymatic activity of the homogenate system. In contrast, SA1‐III and AAT11RI maintained residual concentrations of approximately 75%–80% and 90% at 24 h, respectively. Overall, these results confirm the suitability of the proposed 2D‐HPLC–MS/MS approach for stability assessment of peptides with diverse polarity profiles in complex biological matrices, highlighting the robustness of the chromatographic configuration and IS strategy adopted.

## 4 Conclusion

The stability against dermal proteases is an important aspect to consider in characterizing the pharmacokinetic properties of peptides that must be used topically on the skin (e.g., peptides of cosmeceutical interest). A two‐dimensional HPLC–MS/MS method based on ion trap detection was successfully developed, optimized, and validated for the assessment of peptide stability in HSH. The proposed analytical platform combines online sample cleanup with selective chromatographic separation on a PFP analytical column, enabling reliable quantification of peptides with markedly different physicochemical properties within a single methodological framework. The 2D‐HPLC configuration proved essential for handling complex biological matrices and highly polar analytes, allowing injection of relatively large sample volumes and effective reduction of matrix‐related interferences. The use of a PFP stationary phase further enhanced selectivity and retention control, particularly for structurally related peptide species. Notably, the chromatographic configuration supported the injection of sample solutions containing approximately 30% acetonitrile without compromising analyte focusing during the loading step. This feature enabled reduced sample dilution and minimized unnecessary manipulation, thereby improving detection sensitivity and further highlighting the advantages of the proposed 2D approach over conventional one‐dimensional RP‐HPLC methods. A key strength of the method lies in the IS strategy. The adoption of isomeric peptide analogues (scrambled SA1‐III and the reverse analogue of AAT11RI), sharing identical precursor ions and monitored under the same MS/MS conditions as their corresponding analytes, ensured optimal correction for variability in ionization and fragmentation. In contrast, the use of a structurally unrelated compound (verapamil) as an IS for pal‐KTTKS required dedicated MS/MS acquisition and full chromatographic separation, highlighting the analytical advantages of structurally matched peptide‐based ISs in quantitative bioanalysis. Importantly, the use of isomeric peptide analogues represents a practical and cost‐effective alternative to stable isotope‐labelled standards, particularly in exploratory or screening studies where multiple peptide candidates must be evaluated. Although stable isotope‐labelled analogues remain the benchmark ISs for peptide quantification, the present results highlight the utility of isomeric peptide analogues for exploratory peptide stability studies. The validated method demonstrated satisfactory linearity, sensitivity, precision, accuracy, and minimal MEs across the tested concentration range. The use of an ion trap mass analyzer further contributed to the robustness of the method, providing stable and reproducible product ion distributions under controlled excitation conditions. The characteristic fragmentation behavior of the ion trap platform ensured consistent signal generation and effective control of CID, supporting reliable quantitative performance. Application to dermal stability studies confirmed its suitability for monitoring degradation kinetics of peptides with diverse polarity profiles in HSH. Overall, the proposed 2D‐HPLC–MS/MS approach represents a robust and versatile analytical tool for the quantitative evaluation of peptide stability in complex dermal matrices and may support the preclinical characterization and screening of bioactive peptides intended for topical applications.

## Funding

This study was supported by the Regione Toscana (project: BIOPEPTIDI. Sviluppo di nuovi peptidi biologicamente attivi. PORCREO FESR 2007–2013).

## Supporting information


**TABLE S1:** Gradients used for peptide purifications. Purification steps were performed by flash chromatography on a CombiFlash NextGen300+ Teledyne ISCO instrument with a Teledyne ISCO RediSep Gold 15 g column and by semipreparative chromatography on an HPLC Waters 600 coupled with a Waters UV DAD 2487 with a Sepax Bio‐C18 Column. The solvents used for the reverse‐phase chromatography consisted of H_2_O with 0.1% TFA v/v (solvent A) and acetonitrile with 0.1% TFA v/v (solvent B).
**FIGURE S1:** MS spectrum of pal‐KTTKS.
**FIGURE S2:** MS spectrum of verapamil (IS1).
**FIGURE S3:** MS spectrum of SA1‐IIIsc (IS2).
**FIGURE S4:** MS spectrum of AAT11RI.
**FIGURE S5:** MS spectrum of AAT11‐allD (IS3).
**FIGURE S6:** Collision breakdown plot of pal‐KTTKS.
**FIGURE SF7:** Collision breakdown plot of verapamil.
**FIGURE S8:** Collision breakdown plot of SA1‐III scrambled.
**FIGURE S9:** Collision breakdown plot of AAT11RI.
**FIGURE S10:** Collision breakdown plot of AAT11‐allD.
**FIGURE S11:** MS/MS spectrum of pal‐KTTKS.
**FIGURE S12:** MS/MS spectrum of verapamil (IS1).
**TABLE S2:** Summary of precursor, quantifier, and qualifier ions selected for LC–MS/MS analysis.
**FIGURE S13:** Chromatographic profile of a calibration solution containing pal‐KTTKS and its internal standard (IS1).
**FIGURE S14:** Chromatographic profile of a calibration solution containing SA1‐III and its internal standard (IS2).
**FIGURE S15:** Chromatographic profile of a blank solution containing only IS2.
**FIGURE S16:** Chromatographic profile of a blank solution containing only IS3.
**TABLE S3:** The results of calibration curves obtained for each studied peptide, defined as linear regression parameters (slope and y‐intercept), the determination coefficient (R2), and the estimated LOD and LOQ values.
**FIGURE S17:** Calibration curve of pal‐KTTKS.
**FIGURE S18:** Calibration curve of SA1‐III.
**FIGURE S19:** Calibration curve of AAT11RI.
**FIGURE S20:** Enzymatic degradation of pal‐KTTKS.
**FIGURE S21:** Enzymatic degradation of SA1‐III.
**FIGURE S22:** Enzymatic degradation of AAT11RI.

## Data Availability

The data that supports the findings of this study are available in the supplementary material of this article.
