# Supplementary material for: Application of 2D‐Chromatography System for the Determination of Cosmetic Peptides in Skin Homogenates
Source: J Mass Spectrom. 2026 Jul 23;61(8):e70094. doi: 10.1002/jms.70094 (PMC13395628; doi:10.1002/jms.70094)
Supplement: Supplementary file 1 — TABLE S1: Gradients used for peptide purifications. Purification steps were performed by flash chromatography on a CombiFlash NextGen300+ Teledyne ISCO instrument with a Teledyne ISCO RediSep Gold 15 g column and by semipreparative chromatography on an HPLC Waters 600 coupled with a Waters UV DAD 2487 with a Sepax Bio‐C18 Column. The solvents used for the reverse‐phase chromatography consisted of H2O with 0.1% TFA v/v (solvent A) and acetonitrile with 0.1% TFA v/v (solvent B). FIGURE S1: MS spectrum of pal‐KTTKS. FIGURE S2: MS spectrum of verapamil (IS1). FIGURE S3: MS spectrum of SA1‐IIIsc (IS2). FIGURE S4: MS spectrum of AAT11RI. FIGURE S5: MS spectrum of AAT11‐allD (IS3). FIGURE S6: Collision breakdown plot of pal‐KTTKS. FIGURE SF7: Collision breakdown plot of verapamil. FIGURE S8: Collision breakdown plot of SA1‐III scrambled. FIGURE S9: Collision breakdown plot of AAT11RI. FIGURE S10: Collision breakdown plot of AAT11‐allD. FIGURE S11: MS/MS spectrum of pal‐KTTKS. FIGURE S12: MS/MS spectrum of verapamil (IS1). TABLE S2: Summary of precursor, quantifier, and qualifier ions selected for LC–MS/MS analysis. FIGURE S13: Chromatographic profile of a calibration solution containing pal‐KTTKS and its internal standard (IS1). FIGURE S14: Chromatographic profile of a calibration solution containing SA1‐III and its internal standard (IS2). FIGURE S15: Chromatographic profile of a blank solution containing only IS2. FIGURE S16: Chromatographic profile of a blank solution containing only IS3. TABLE S3: The results of calibration curves obtained for each studied peptide, defined as linear regression parameters (slope and y‐intercept), the determination coefficient (R2), and the estimated LOD and LOQ values. FIGURE S17: Calibration curve of pal‐KTTKS. FIGURE S18: Calibration curve of SA1‐III. FIGURE S19: Calibration curve of AAT11RI. FIGURE S20: Enzymatic degradation of pal‐KTTKS. FIGURE S21: Enzymatic degradation of SA1‐III. FIGURE S22: Enzymatic degradation of AAT11RI. [file JMS-61-e70094-s001.docx]

# *Application of 2D-chromatography system for the determination of cosmetic peptides in skin homogenates*

Fosca Errante^a,b^, Marco Pallecchi^a^, Marta Menicatti^a^, Lisa Giovannelli^a^, Paolo Rovero^a,b^ and Gianluca Bartolucci^a^.

a) Department of Neurofarba (Department of Neurosciences, Psychology, Drug Research and Child Health), University of Florence, Italy

b) Interdepartmental Research Unit of Peptides and Proteins Chemistry and Biology, University of Florence, Italy

Fosca Errante: [fosca.errante@unifi.it](mailto:fosca.errante@unifi.it), ORCID ID 0000-0001-7790-9886

Marta Menicatti: [marta.menicatti@unifi.it](mailto:marta.menicatti@unifi.it), ORCID ID 0000-0001-8302-7644

Marco Pallecchi: [marco.pallecchi@unifi.it](mailto:marco.pallecchi@unifi.it), ORCID ID 0000-0002-0057-6531

Lisa Giovannelli: [lisa.giovannelli@unifi.it](mailto:lisa.giovannelli@unifi.it), ORCID ID 0000-0002-4027-4693

Paolo Rovero: [paolo.rovero@unifi.it](mailto:paolo.rovero@unifi.it), ORCID ID 0000-0001-9577-5228

Corresponding author Gianluca Bartolucci: [gianluca.bartolucci@unifi.it](mailto:gianluca.bartolucci@unifi.it), Via Ugo Schiff 6, Department of Neurofarba, University of Florence, Sesto Fiorentino (FI) 50019, Italy, ORCID ID 0000-0002-5631-8769

**Supporting Information**

Table ST1. Gradients used for peptide purifications. Purification steps were performed by flash chromatography on a CombiFlash NextGen300+ Teledyne ISCO instrument with a Teledyne ISCO RediSep Gold 15g column, and by semipreparative chromatography on an HPLC Waters 600 coupled with a Waters UV DAD 2487 with a Sepax Bio-C18 Column. The solvents used for the reverse-phase chromatography consisted of H_2_O with 0.1% TFA v/v (solvent A) and acetonitrile with 0.1% TFA v/v (solvent B).

| **Compound** | **Flash Chromatography** | **Semi-preparative** |
| --- | --- | --- |
| pal-KTTKS | 20-80 % B in 16 min | 40-80 % B in 30 min |
| SA1-III | 10-50 % B in 16 min | 10-40 % B in 30 min |
| SA1-IIIsc (IS2) | 10-50 % B in 16 min | 10-40 % B in 30 min |
| AAT11RI | 05-40 % B in 16 min | 05-40 % B in 30 min |
| AAT11-allD (IS3) | 05-40 % B in 16 min | 05-40 % B in 30 min |

**
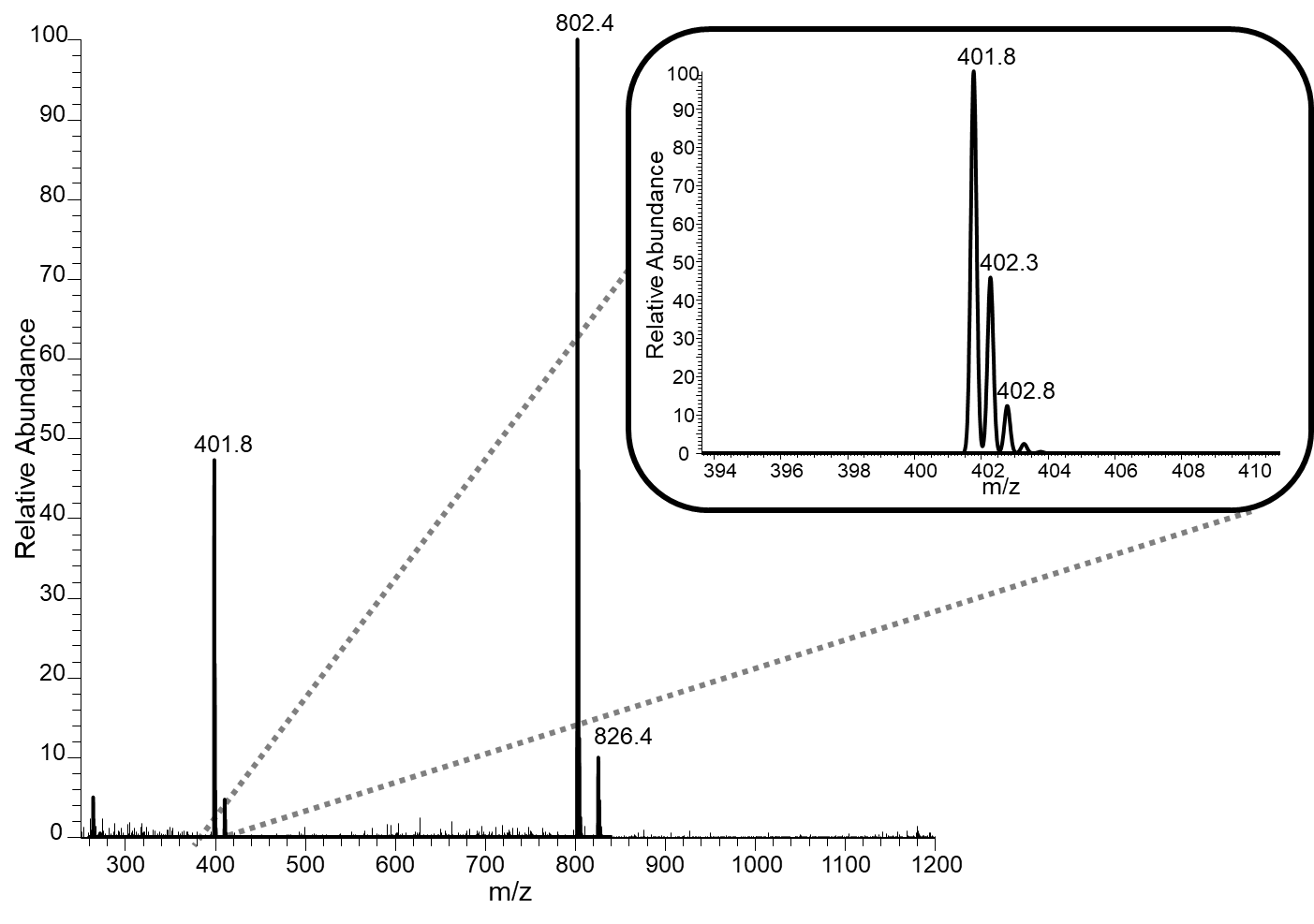
**

**SF1:** MS spectrum of pal-KTTKS

**
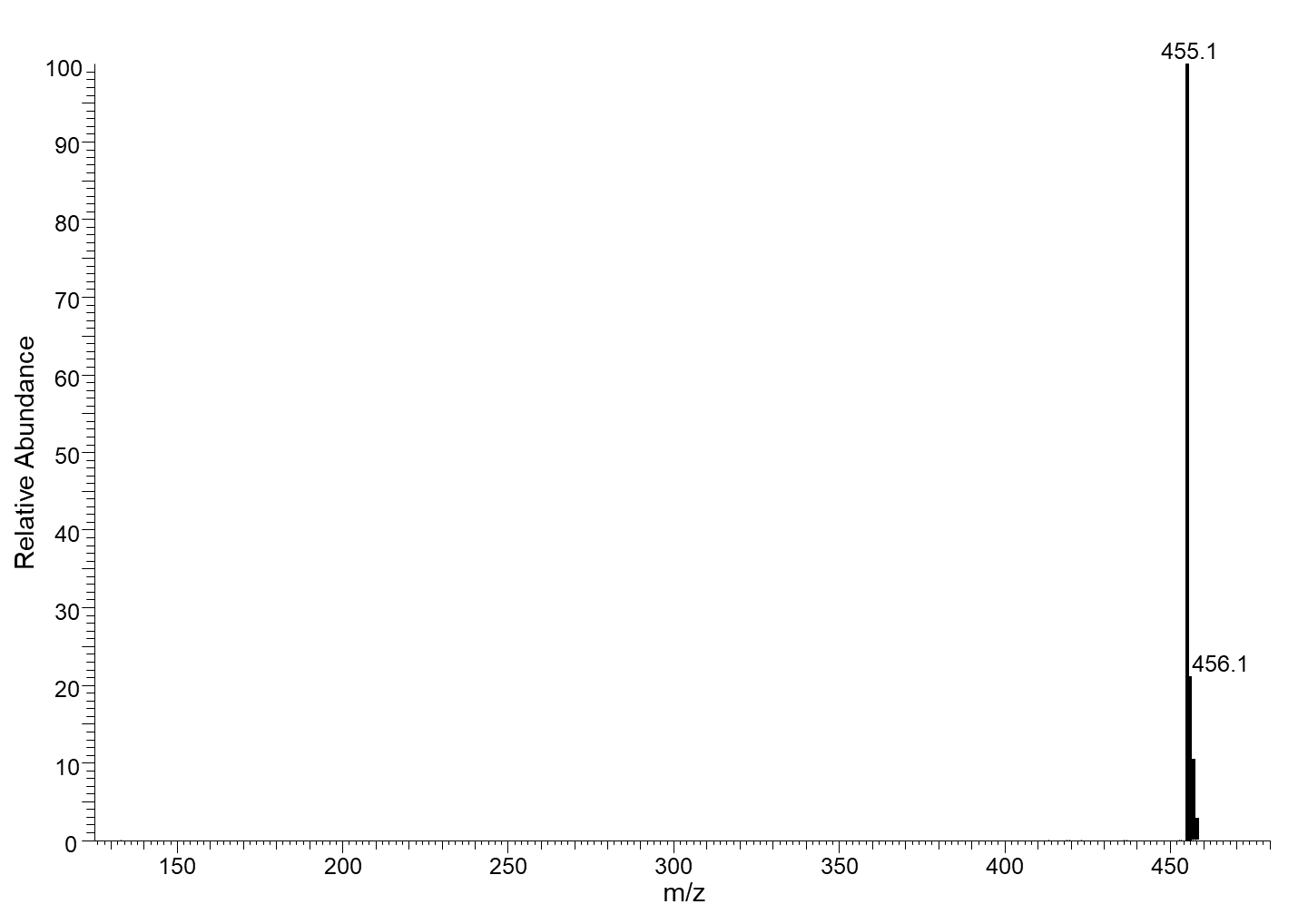
**

**SF2:** MS spectrum of verapamil (IS1)

**
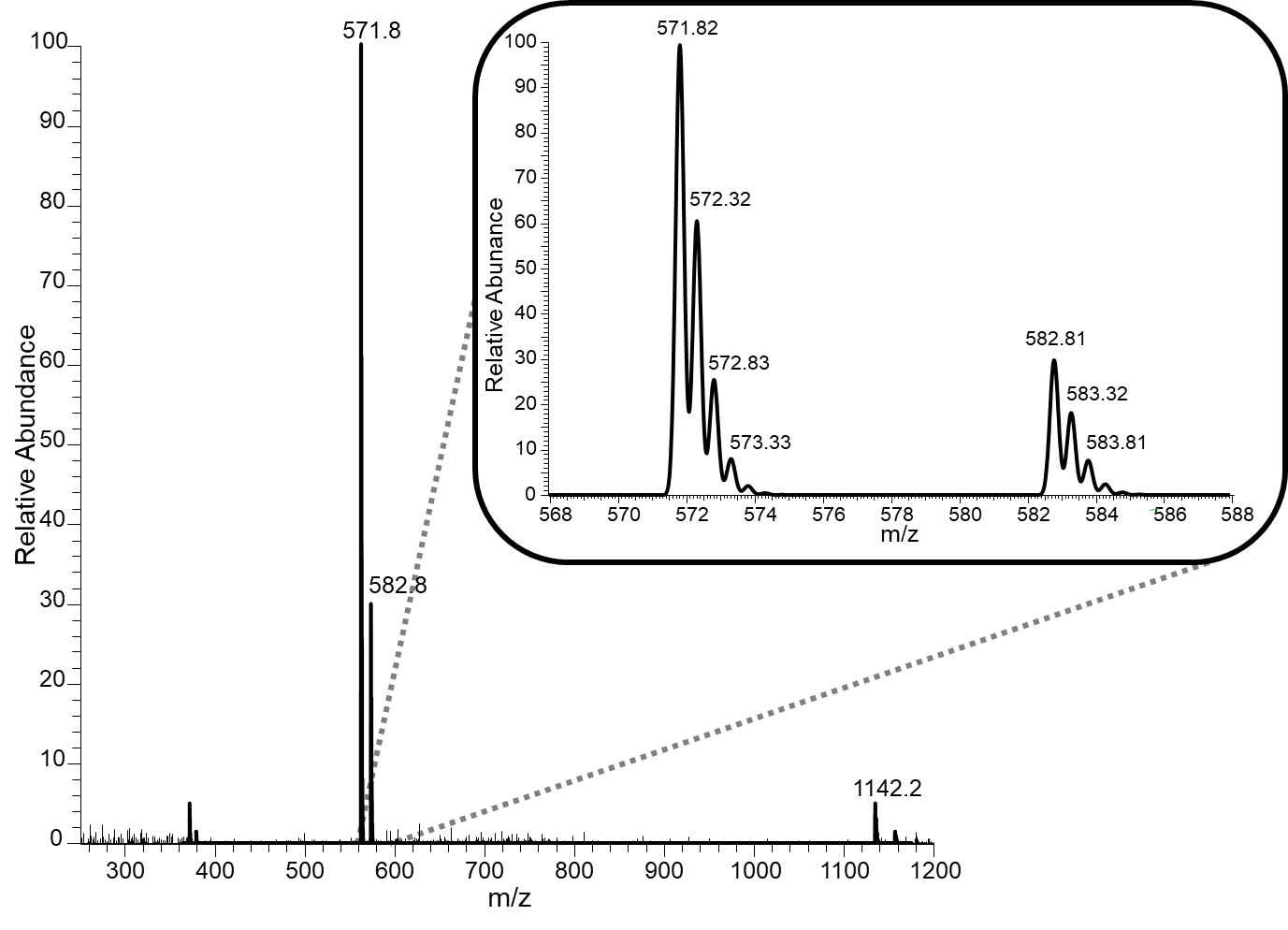
**

**SF3:** MS spectrum of SA1-III_sc_ (IS2)

**
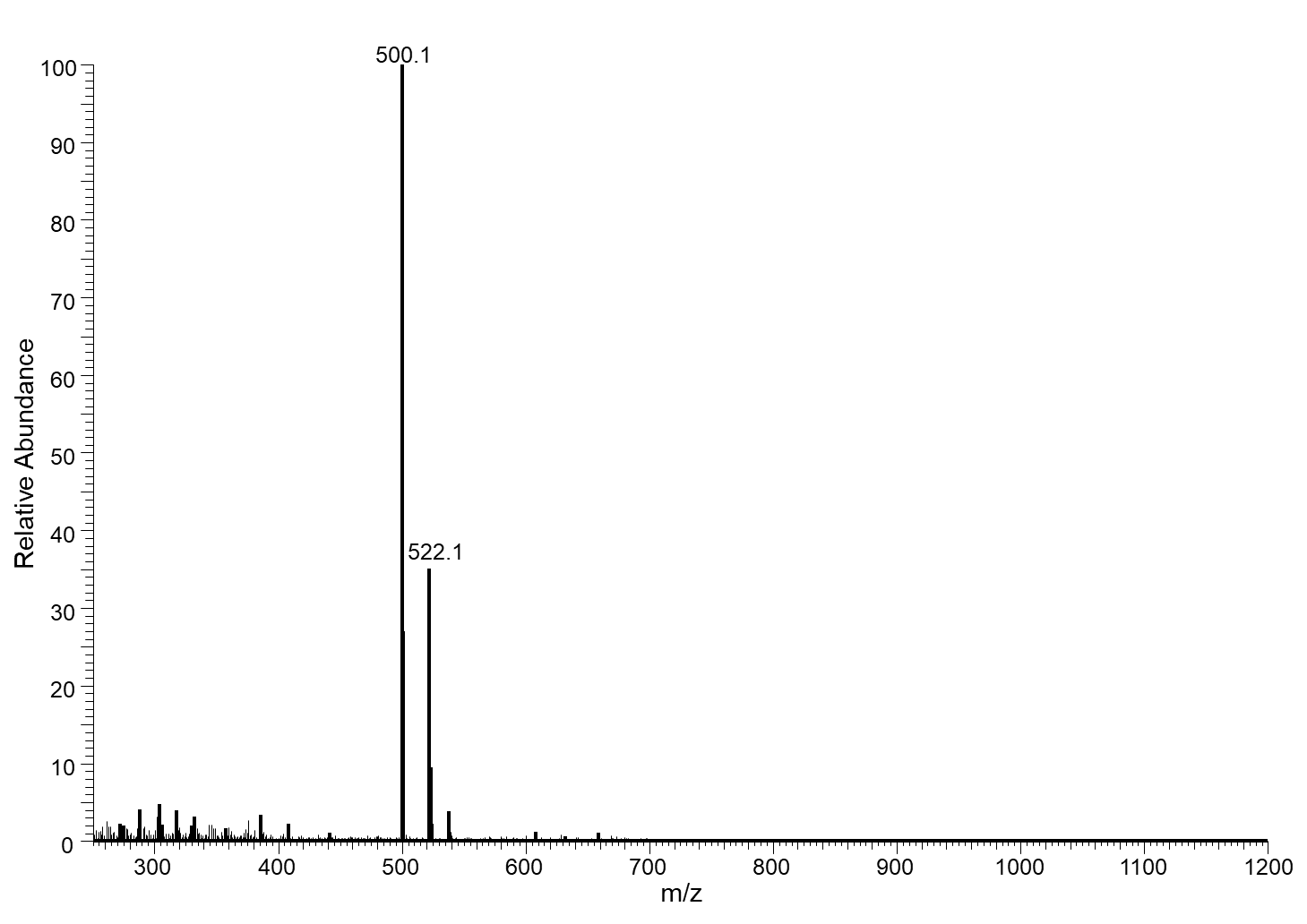
**

**SF4:** MS spectrum of AAT11RI

**
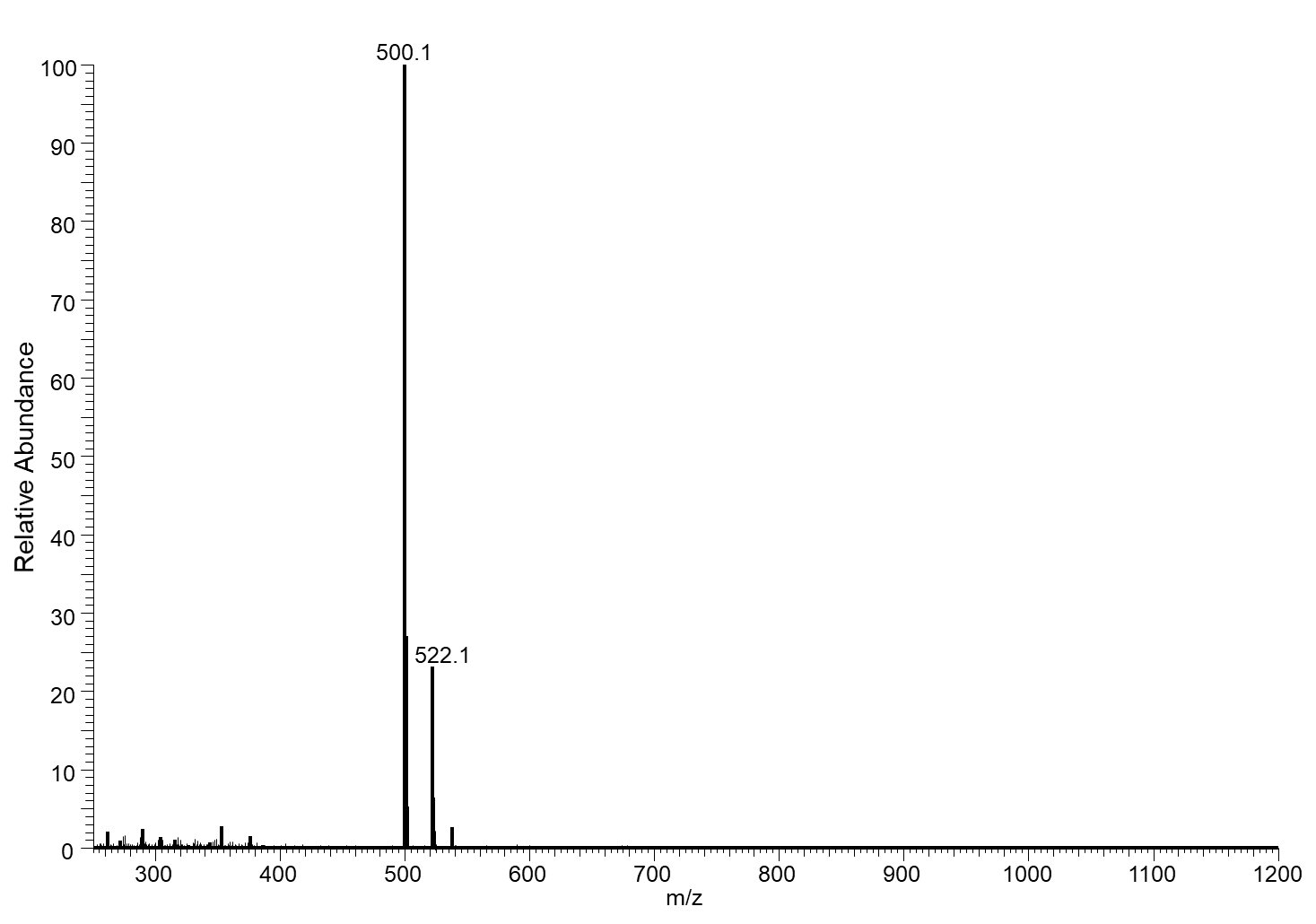
**

**SF5:** MS spectrum of AAT11-allD (IS3)

**
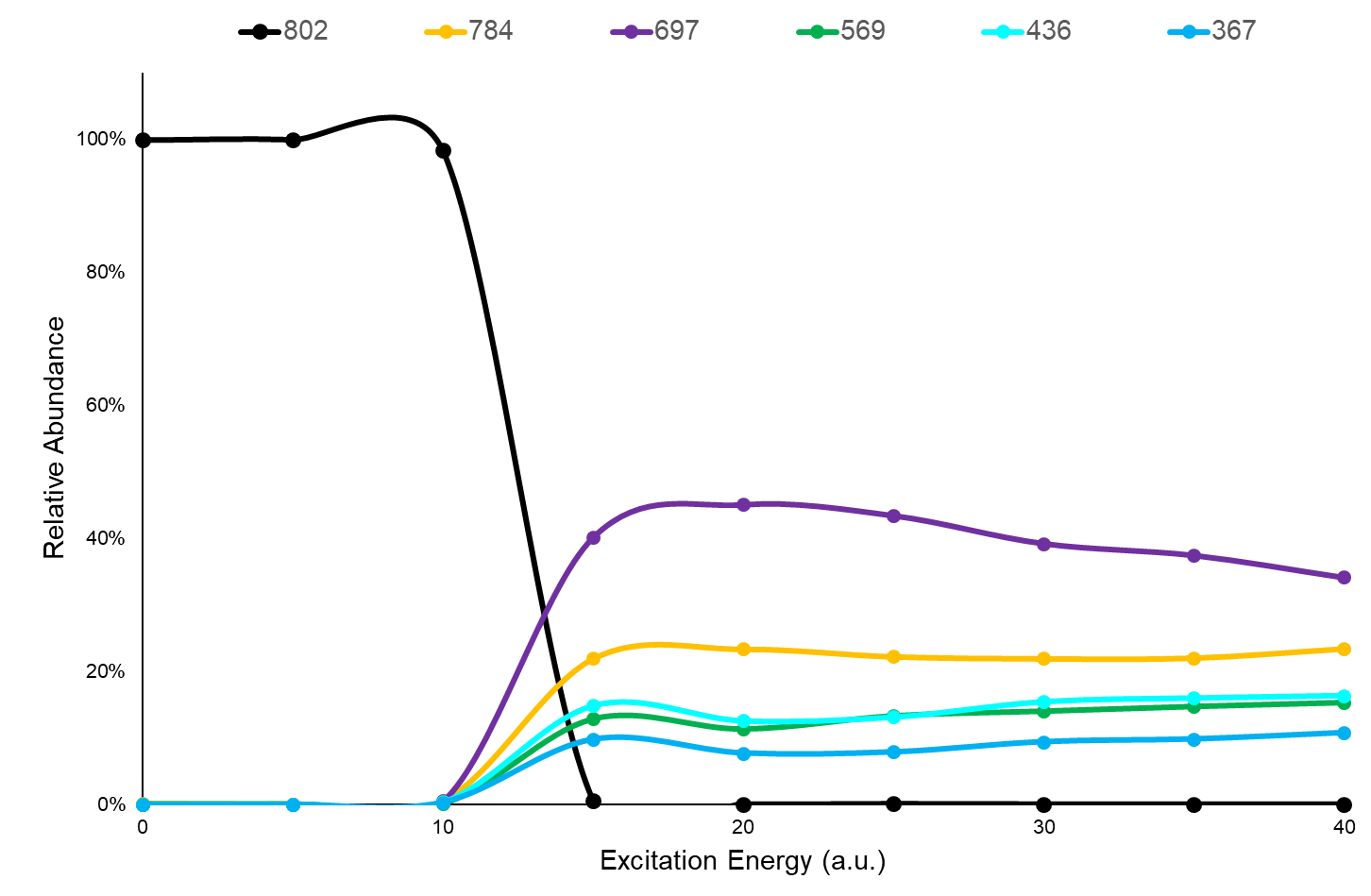
**

**SF6:** Collision Breakdown plot of pal-KTTKS

**
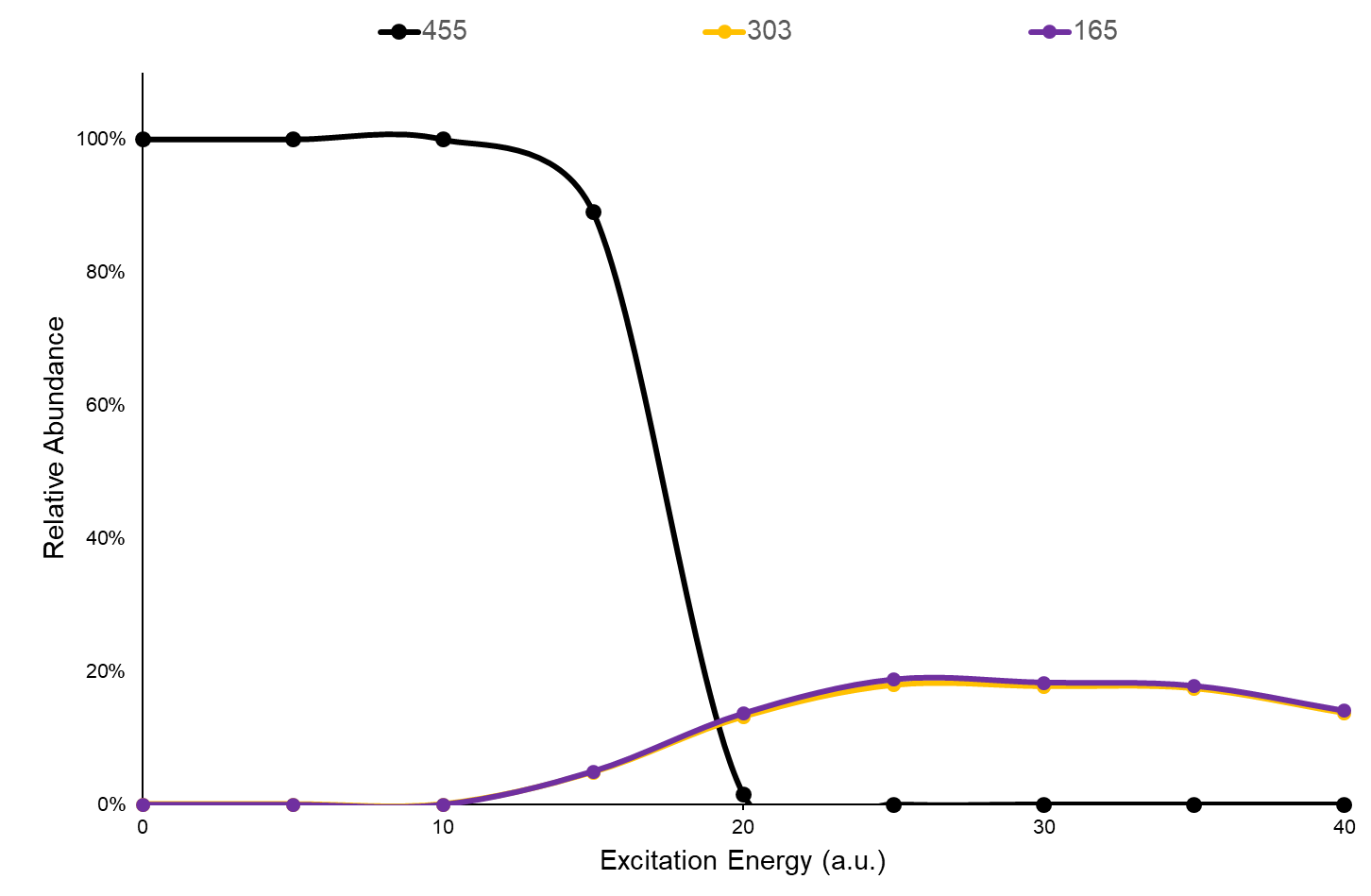
**

**SF7:** Collision Breakdown plot of verapamil

**
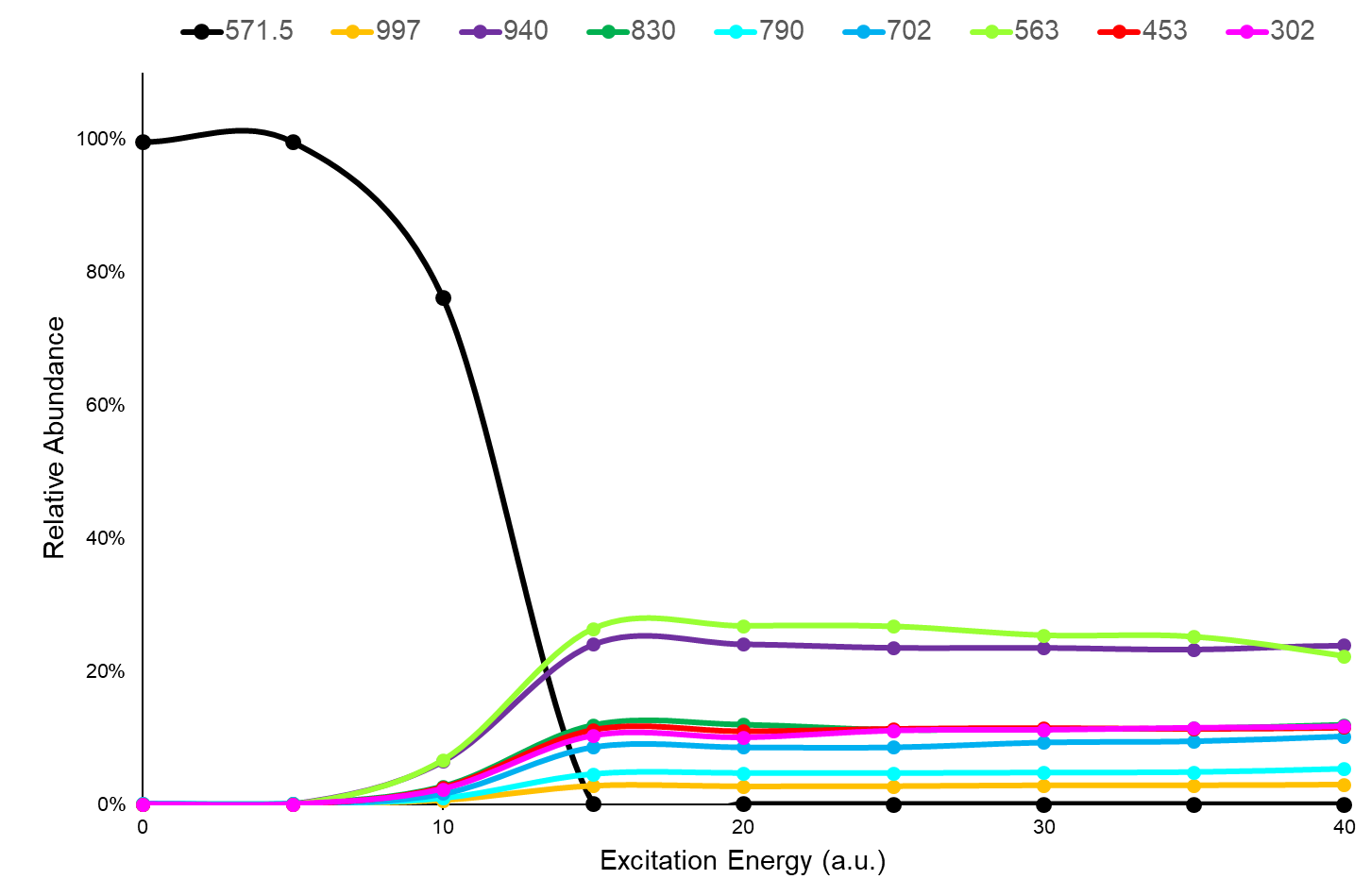
**

**SF8:** Collision Breakdown plot of SA1-III scrambled

**
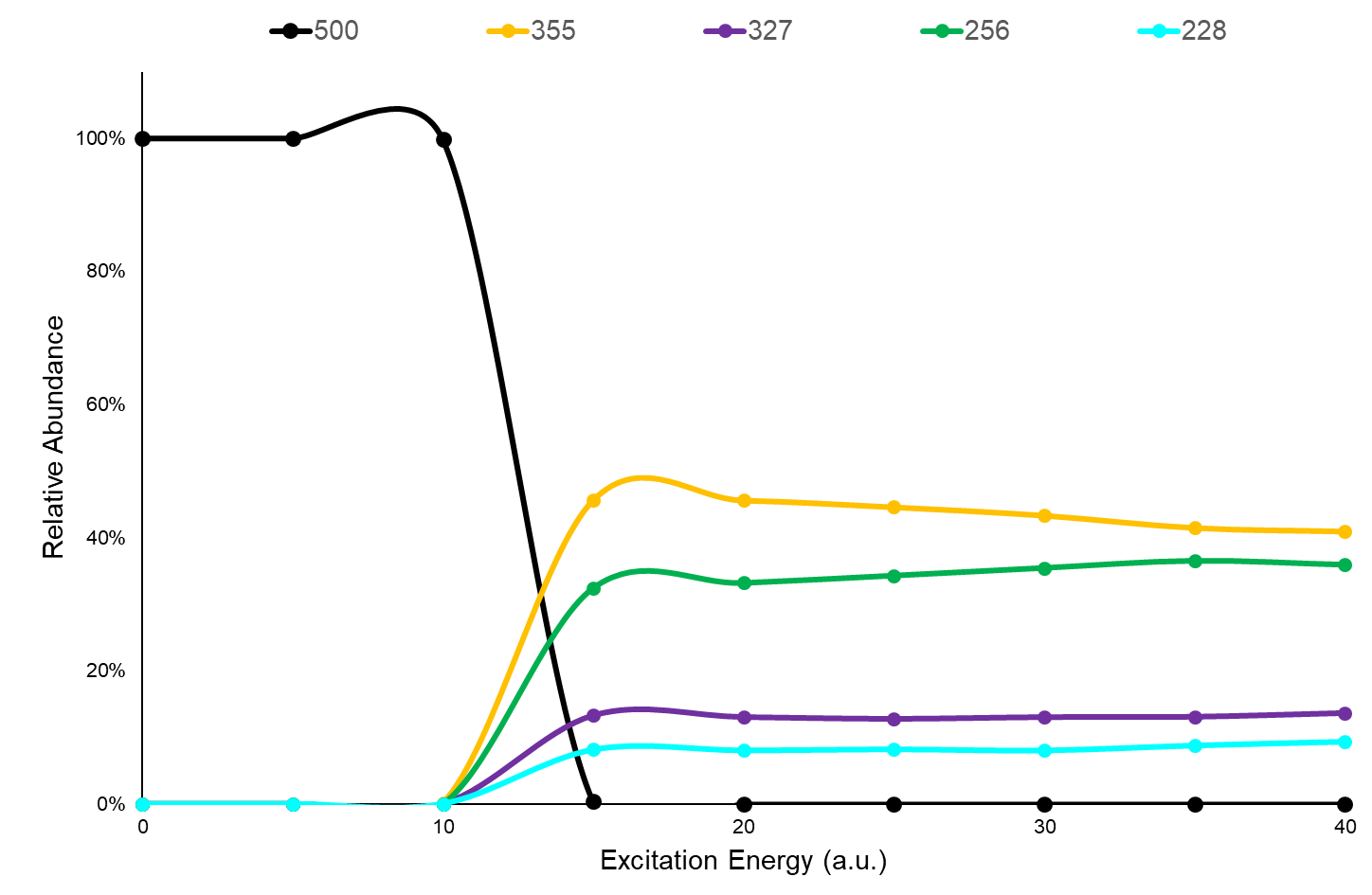
**

**SF9:** Collision Breakdown plot of AAT11RI

**
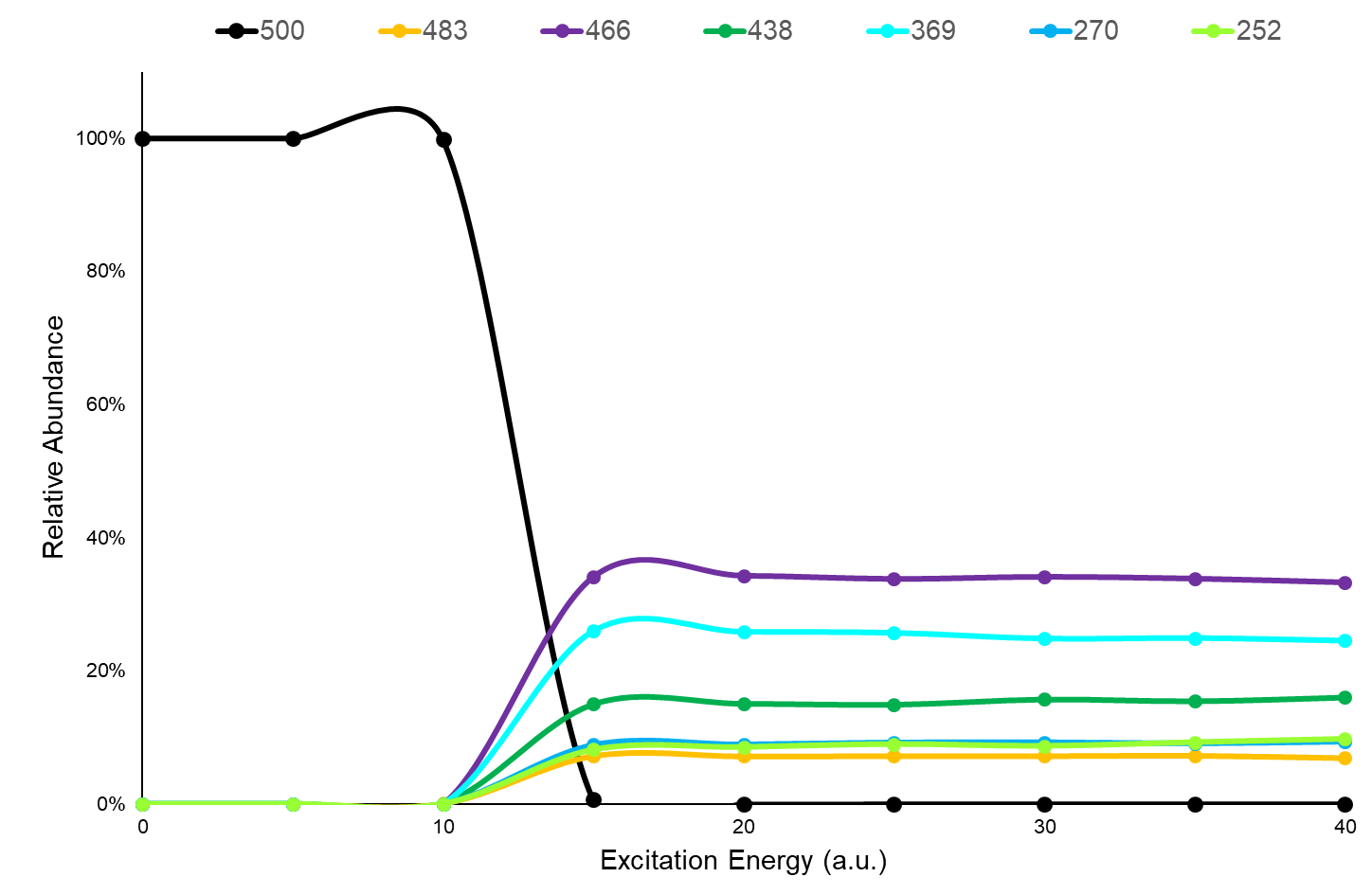
**

**SF10:** Collision Breakdown plot of AAT11-allD

**
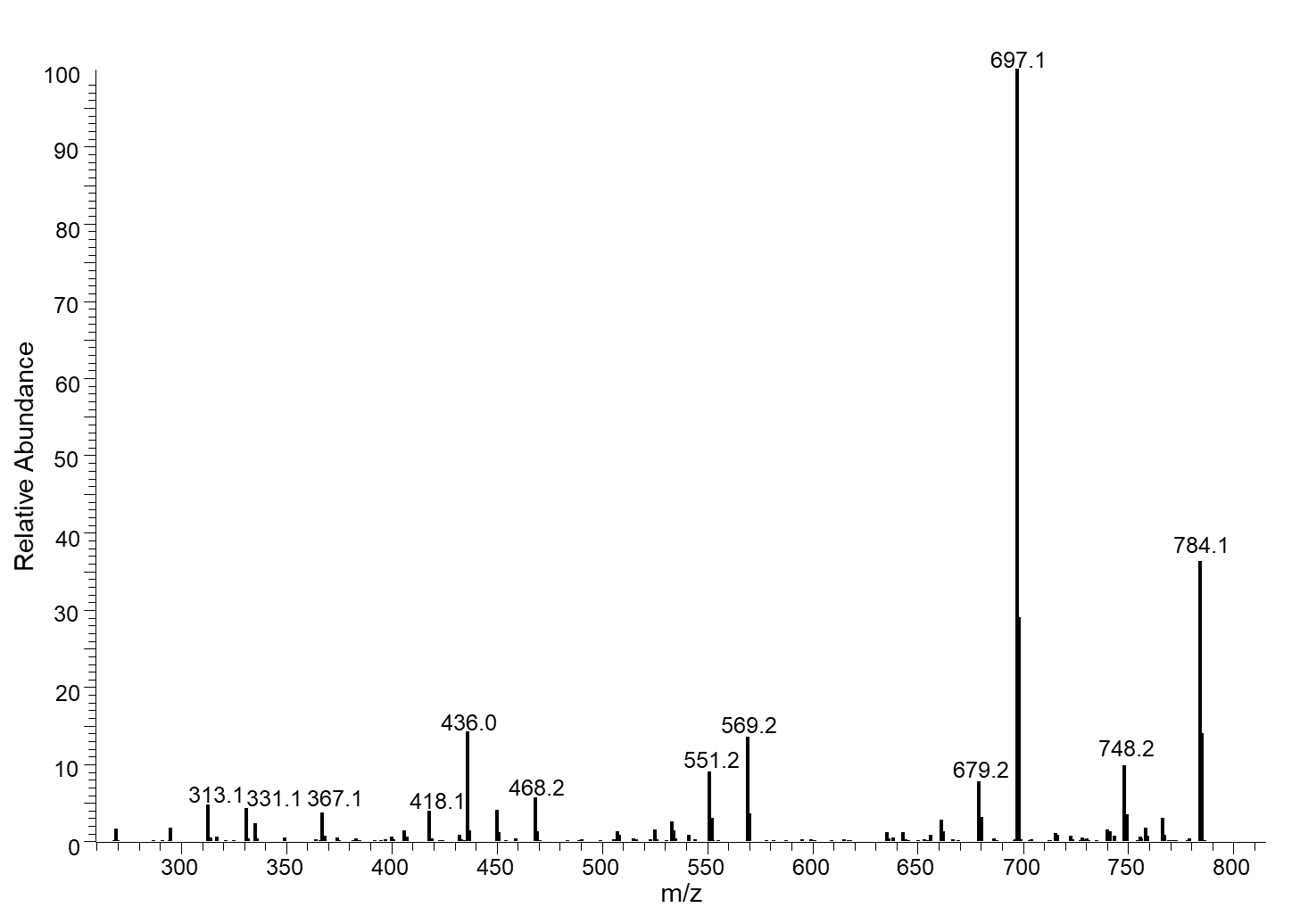
**

**SF11:** MS/MS spectrum of pal-KTTKS

**
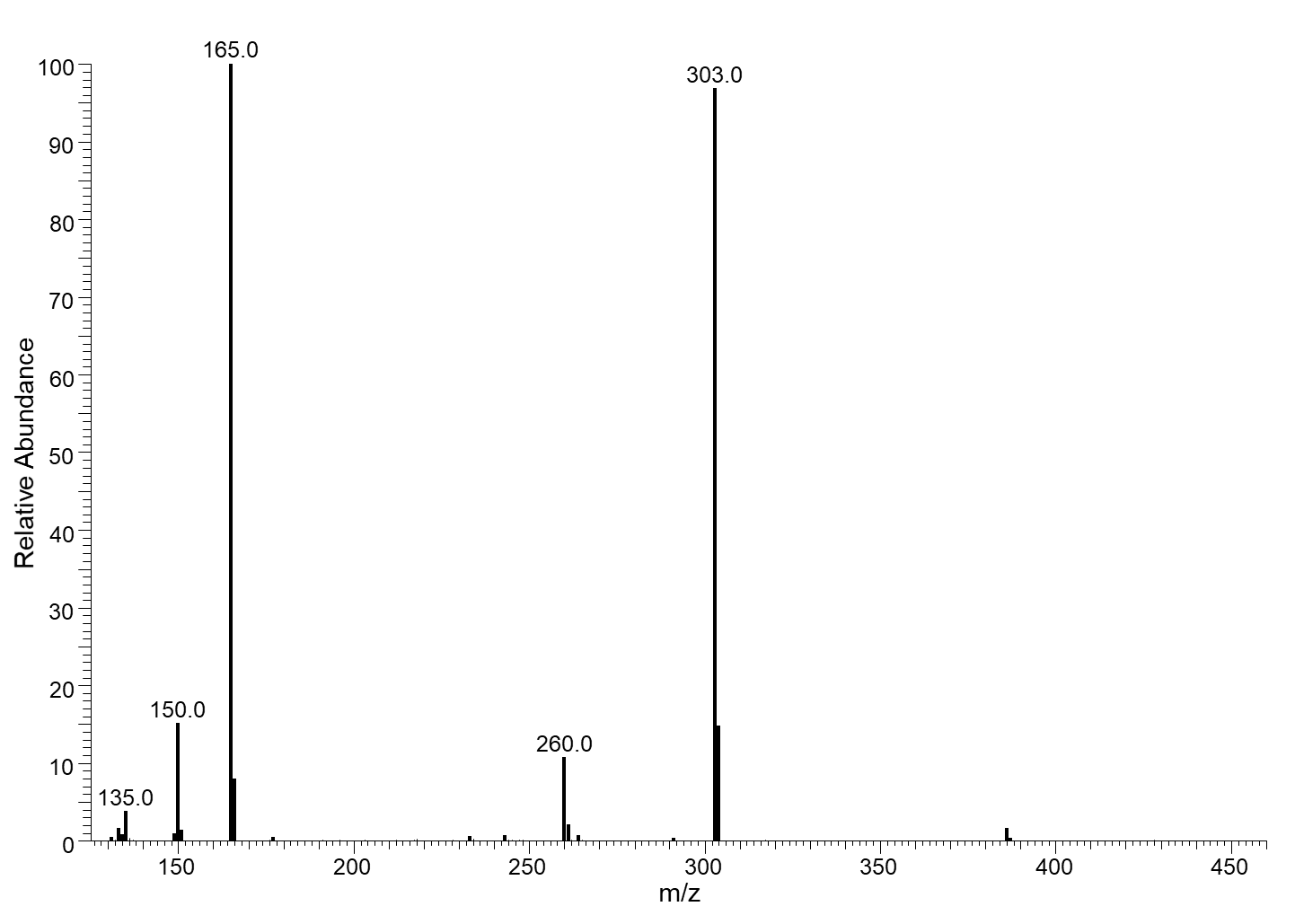
**

**SF12:** MS/MS spectrum of verapamil (IS1)

Table ST2. Summary of precursor, quantifier, and qualifier ions selected for LC–MS/MS analysis

| **Compound** | **Precursor ion**  **(m/z)** | **Charge state** | **Quantifier ion (m/z)** | **Qualifier ion (m/z)** |
| --- | --- | --- | --- | --- |
| pal-KTTKS | 802.4 | 1+ | 697 | 784 |
| Verapamil (IS1) | 455.4 | 1+ | 165 | 303 |
| SA1-III | 571.8 | 2+ | 997 | 980 |
| SA1-IIIsc (IS2) | 571.8 | 2+ | 940 | 923 |
| AAT11RI | 500.1 | 1+ | 355 | 256 |
| AAT11-allD (IS3) | 500.1 | 1+ | 369 | 270 |

**
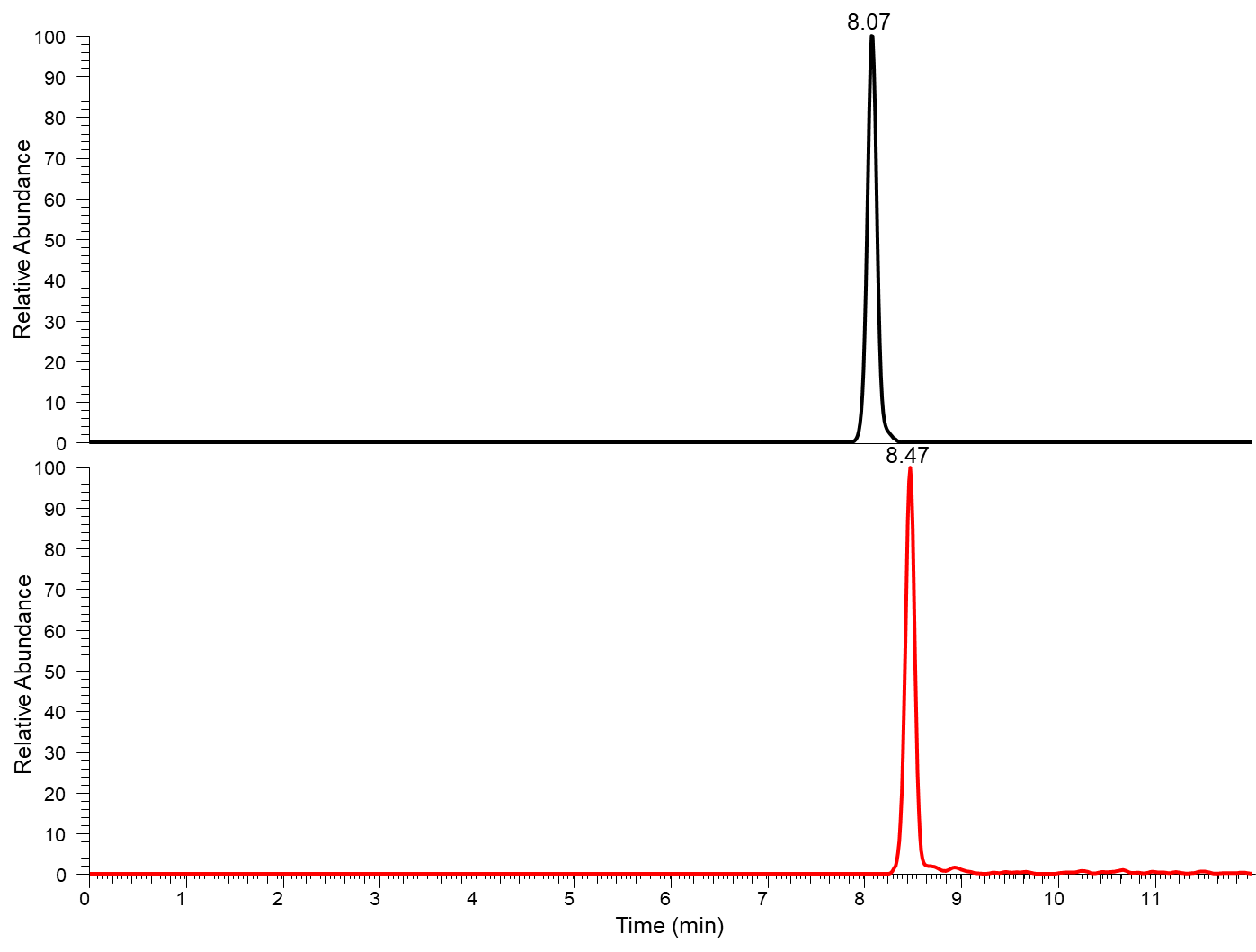
**

**SF13:** Chromatographic profile of a calibration solution containing pal-KTTKS and its internal standard (IS1).


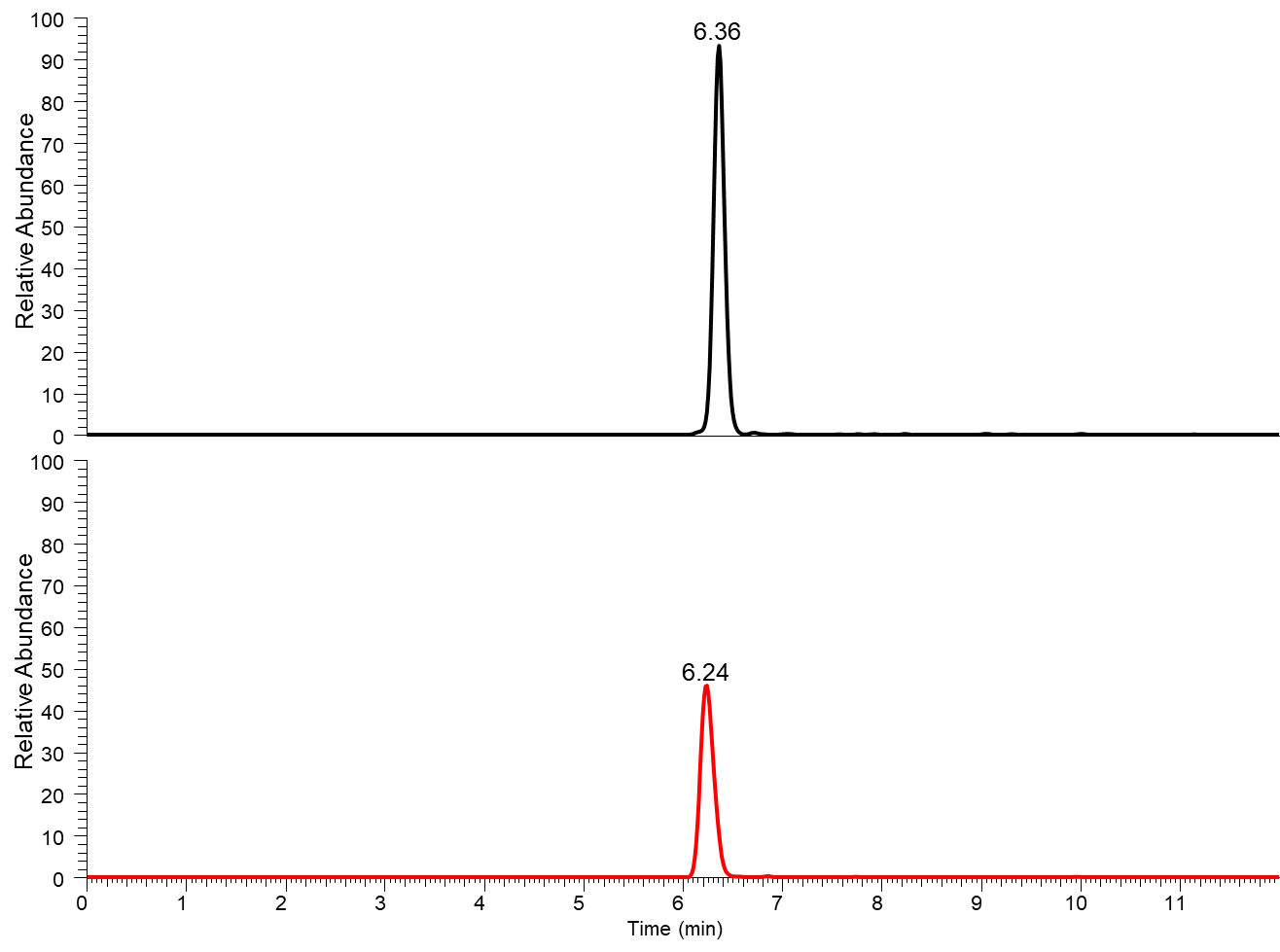


**SF14:** Chromatographic profile of a calibration solution containing SA1-III and its internal standard (IS2).

**
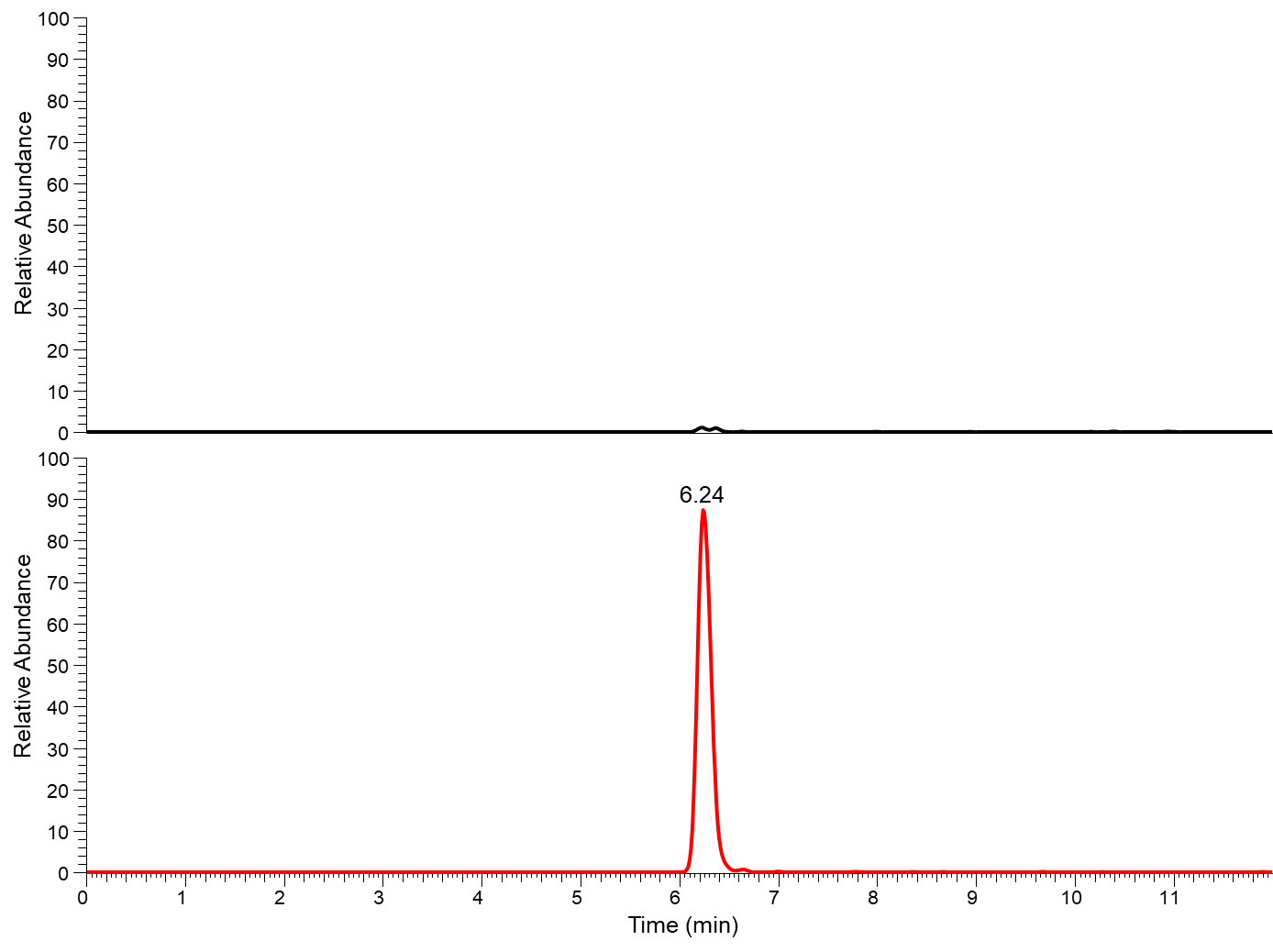
**

**SF15:** Chromatographic profile of a Blank solution containing only IS2


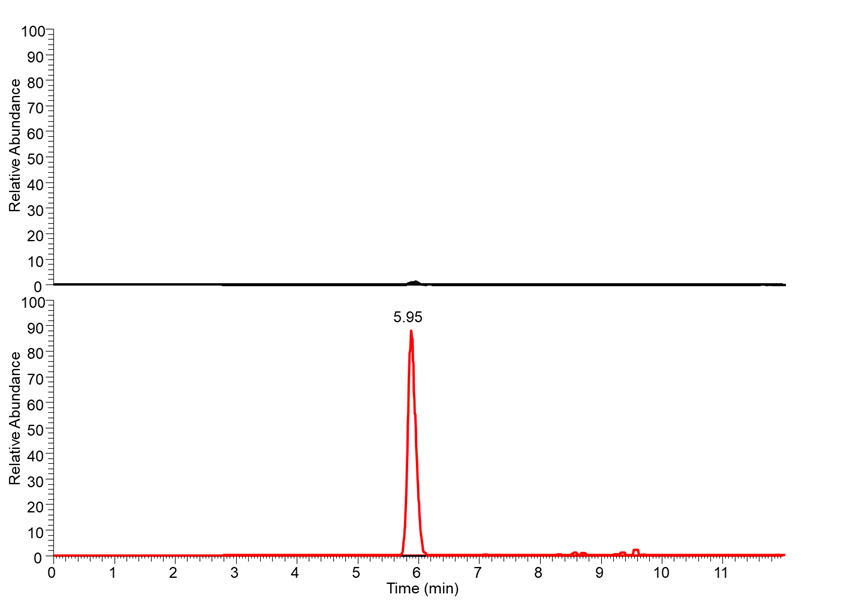


**SF16:** Chromatographic profile of a Blank solution containing only IS3

**ST3:** The results of calibration curves obtained for each studied peptide, defined as linear regressions parameters (slope and y-intercept), the determination coefficient (R^2^) and the estimated LOD and LOQ values.

|  | Slope (PAR/μM) | Intercept (PAR) | (R^2^) | LOD (μM) | LOQ (μM) |
| --- | --- | --- | --- | --- | --- |
| pal-KTTKS | 0.20 | -0.01 | 0.992 | 0.03 | 0.10 |
| SA1-III | 2.06 | -0.02 | 0.996 | 0.03 | 0.10 |
| AAT11RI | 4.75 | 0.06 | 0.998 | 0.02 | 0.05 |

**
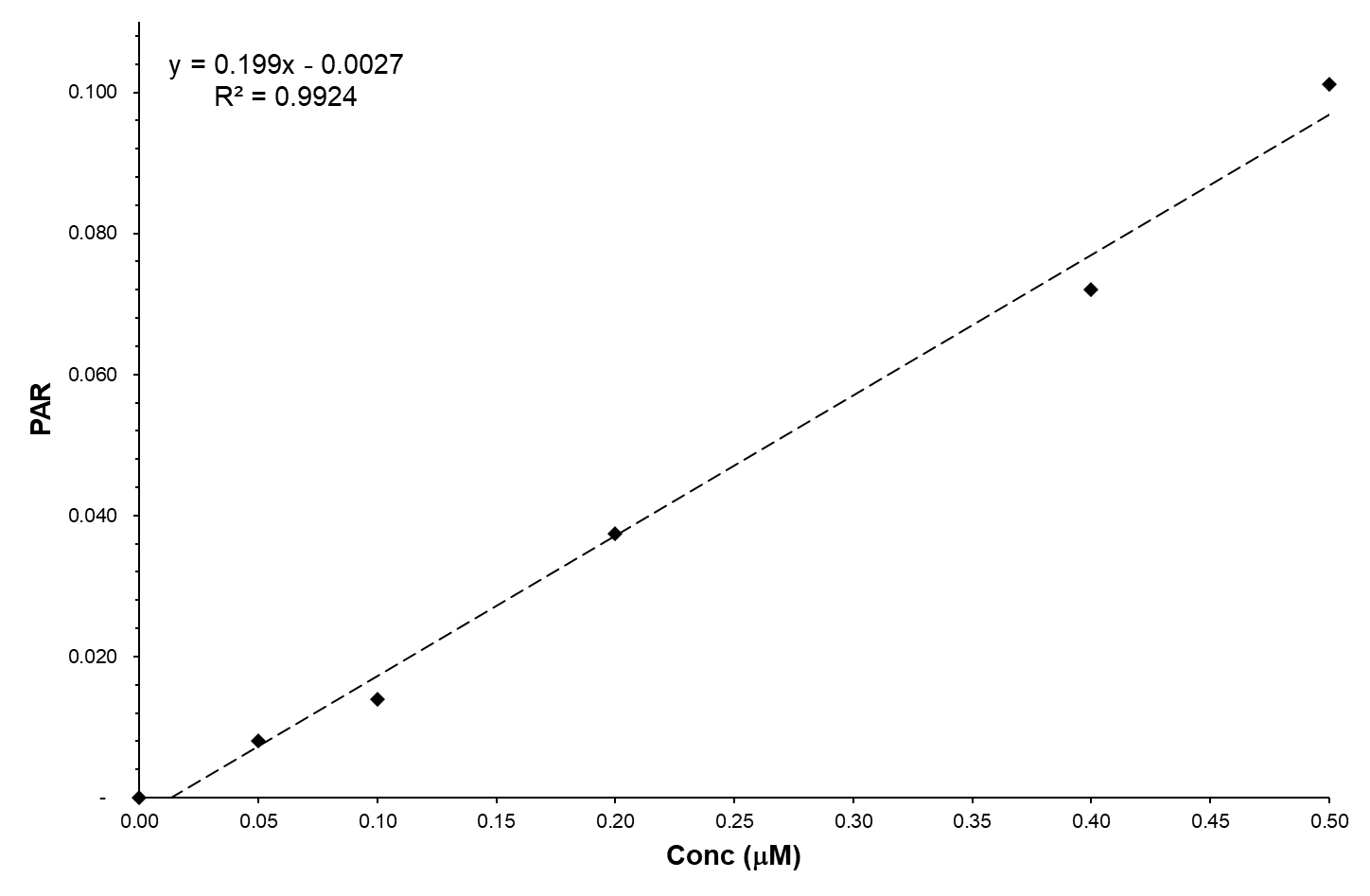
**

**SF17:** Calibration curve of pal-KTTKS

**
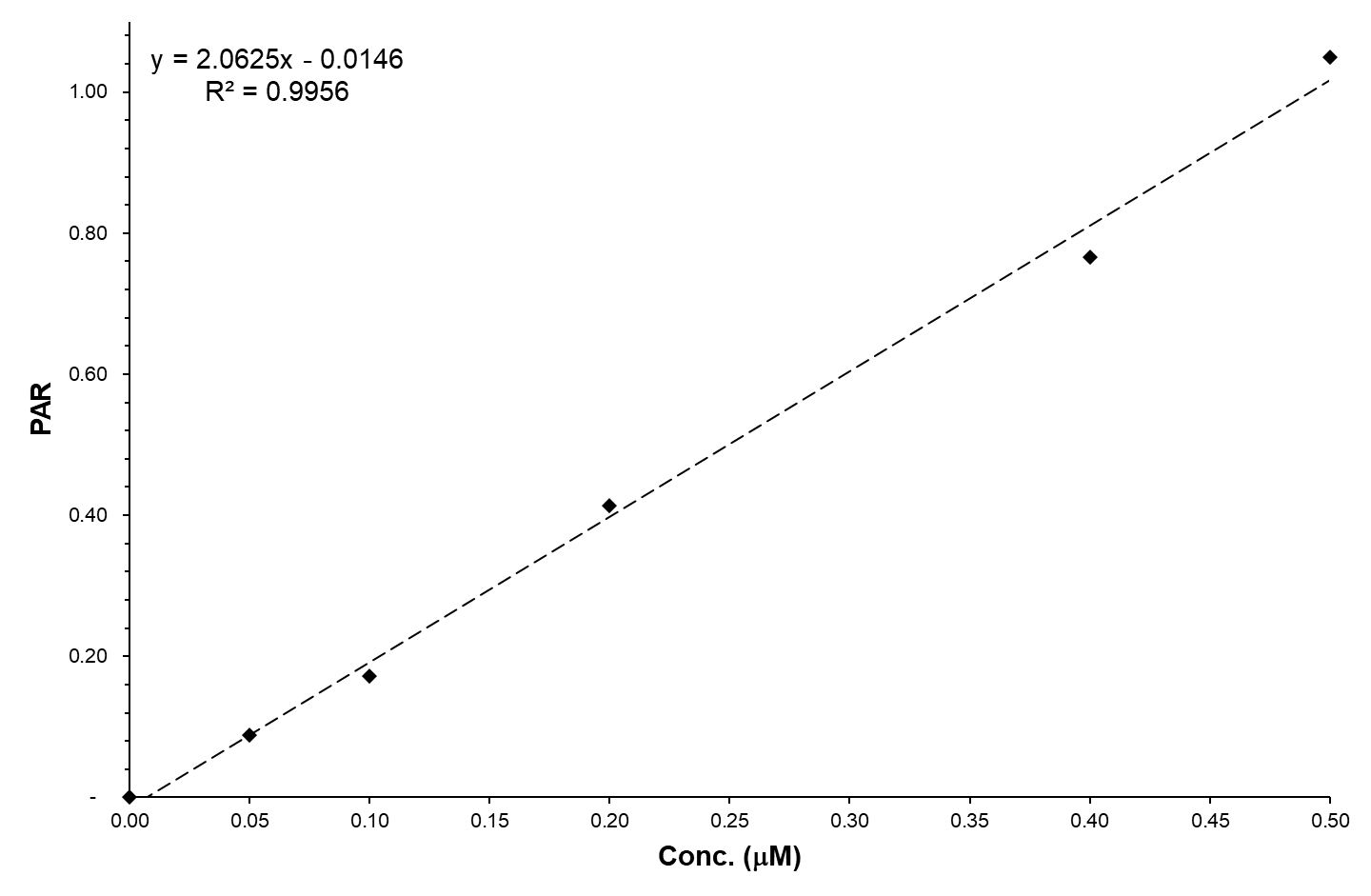
**

**SF18:** Calibration curve of SA1-III


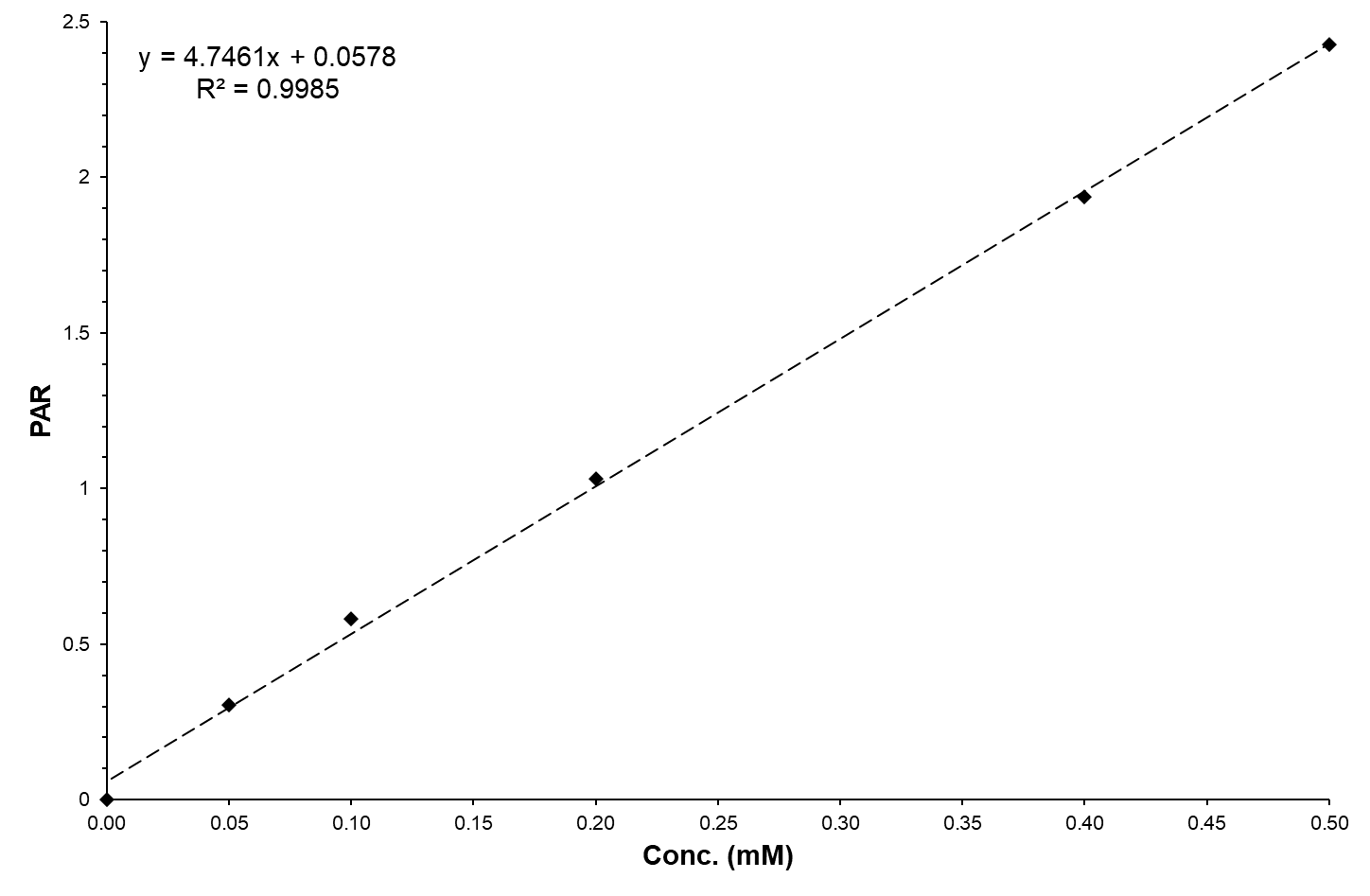


**SF19:** Calibration curve of AAT11RI

**
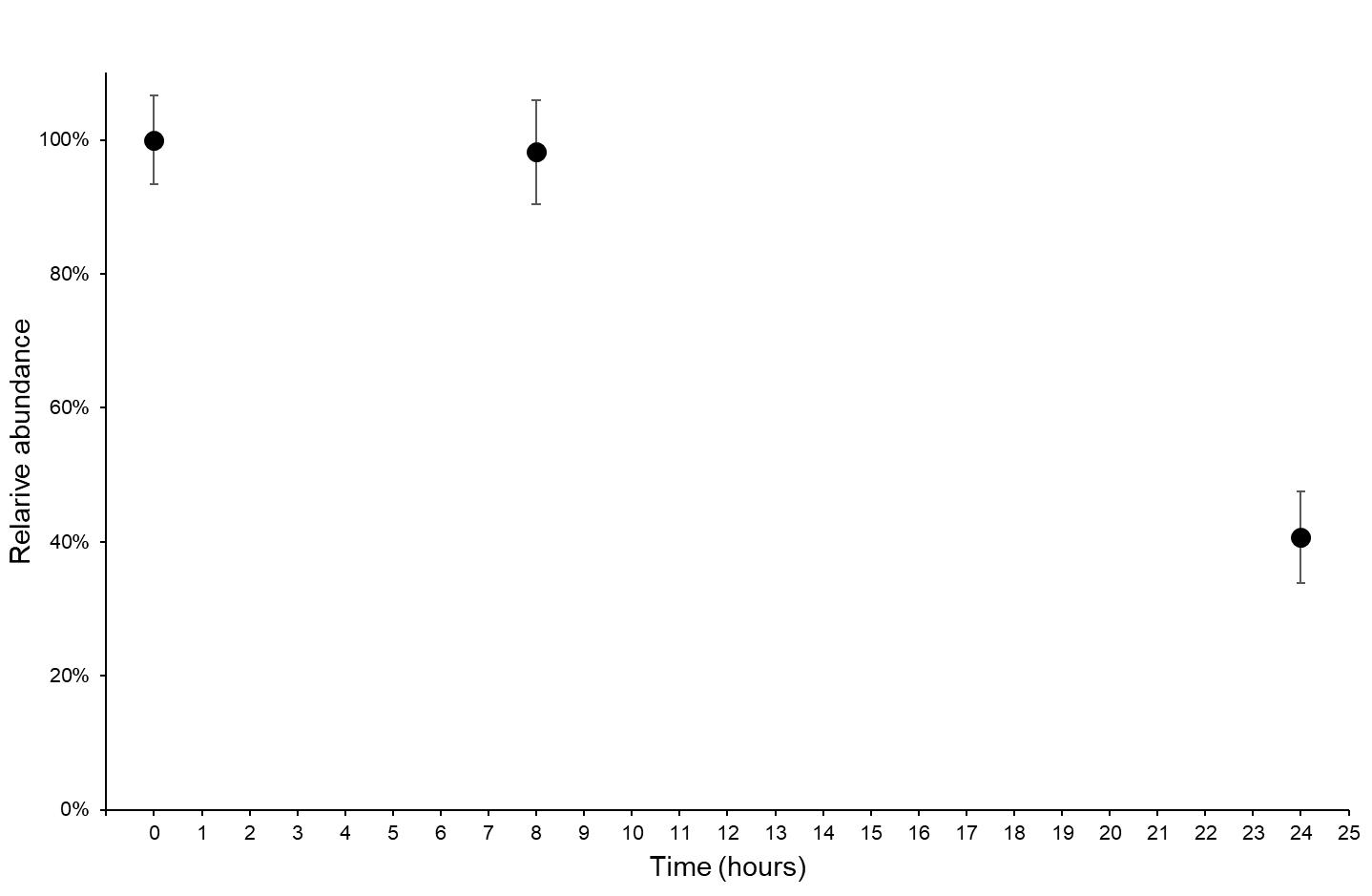
**

**SF20:** Enzymatic degradation of pal-KTTKS

**
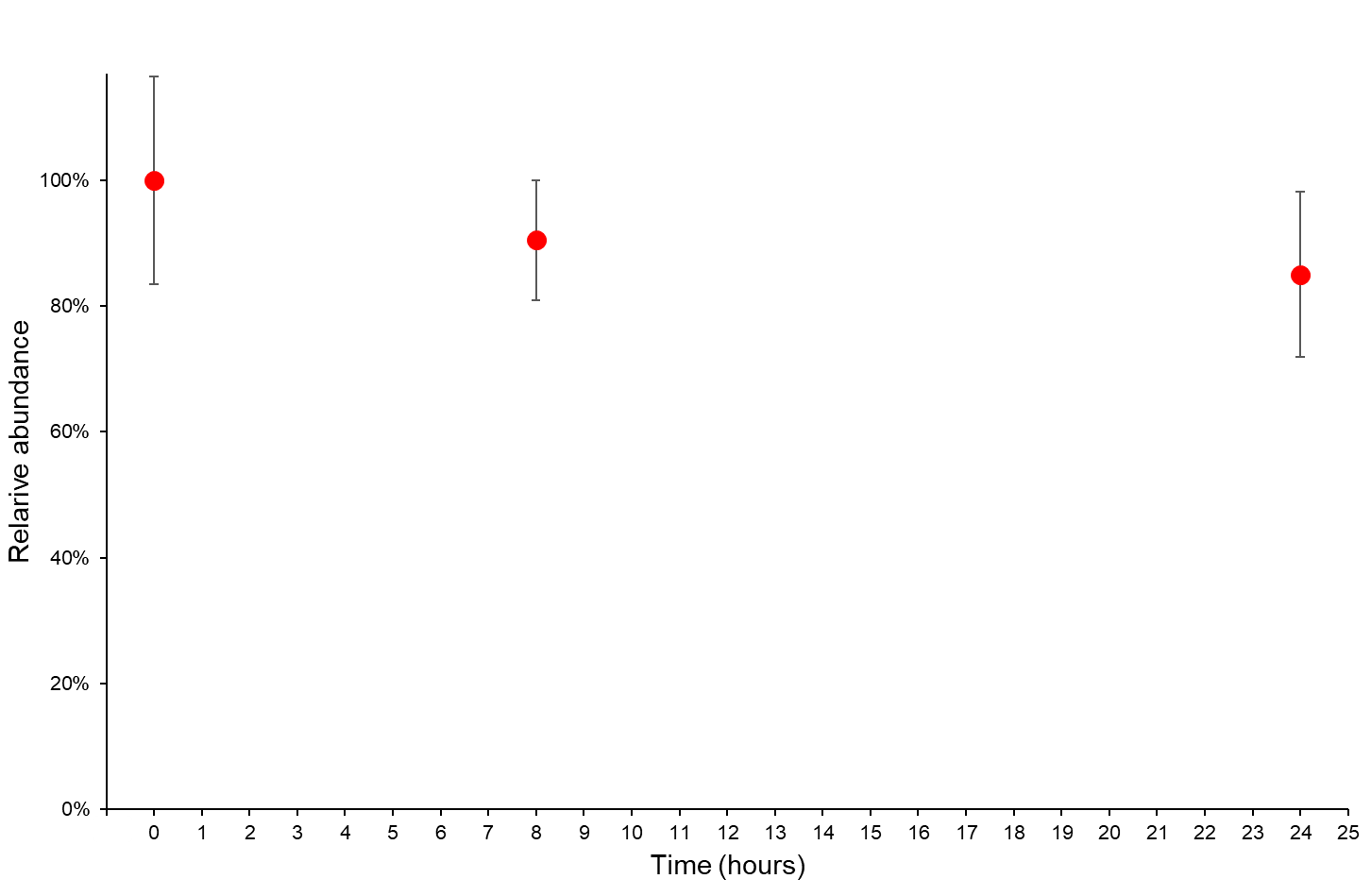
**

**SF21:** Enzymatic degradation of SA1-III

**
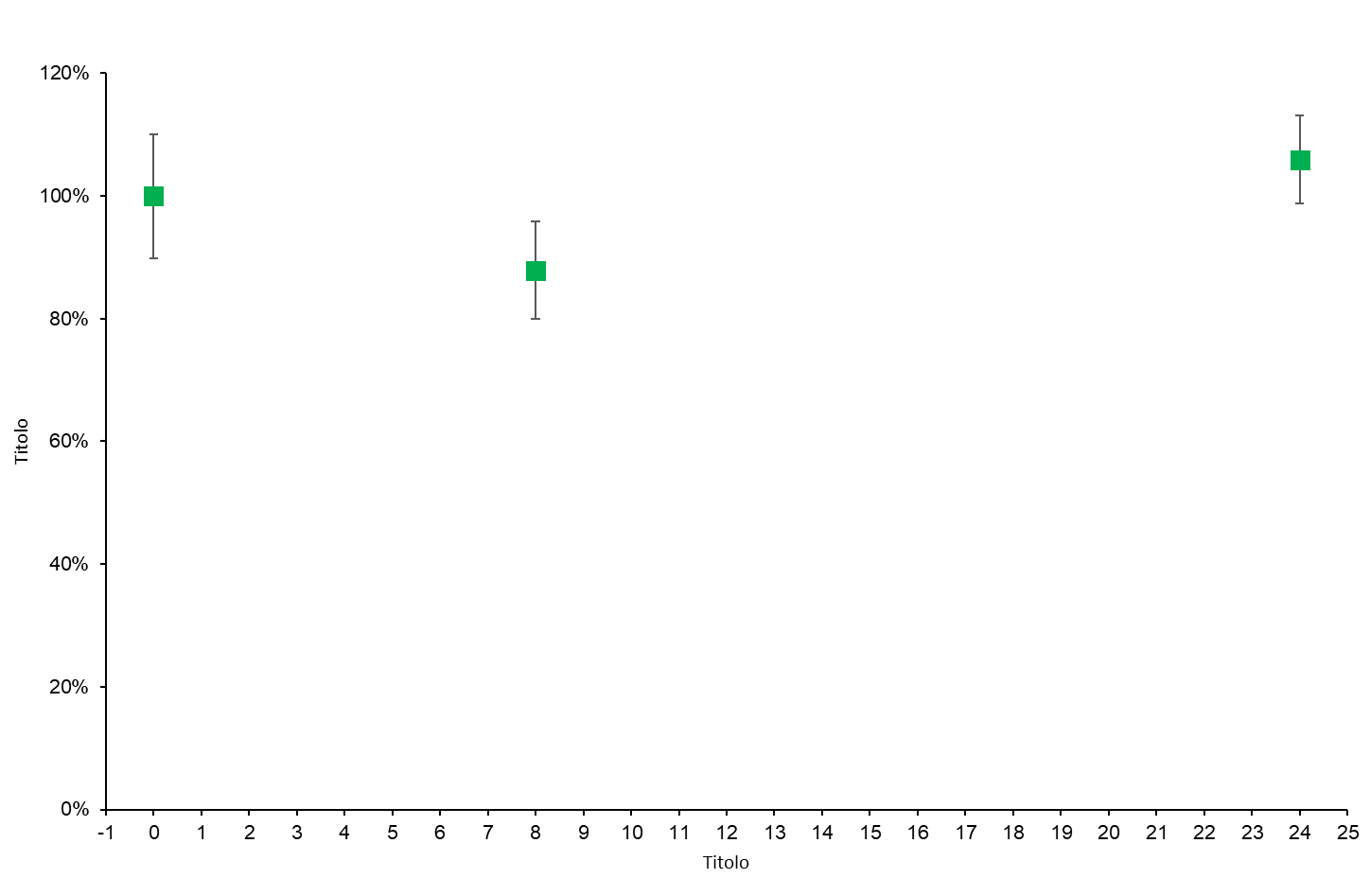
**

**SF22:** Enzymatic degradation of AAT11RI
